# Locomotor Activity and Body Temperature Patterns over a Temperature Gradient in the Highveld Mole-Rat (*Cryptomys hottentotus pretoriae*)

**DOI:** 10.1371/journal.pone.0169644

**Published:** 2017-01-10

**Authors:** Meghan Haupt, Nigel C. Bennett, Maria K. Oosthuizen

**Affiliations:** Department of Zoology & Entomology, University of Pretoria, Pretoria, South Africa; Kent State University, UNITED STATES

## Abstract

African mole-rats are strictly subterranean mammals that live in extensive burrow systems. High humidity levels in the burrows prevent mole-rats from thermoregulating using evaporative cooling. However, the relatively stable environment of the burrows promotes moderate temperatures and small daily temperature fluctuations. Mole-rats therefore display a relatively wide range of thermoregulation abilities. Some species cannot maintain their body temperatures at a constant level, whereas others employ behavioural thermoregulation. Here we test the effect of ambient temperature on locomotor activity and body temperature, and the relationship between the two parameters, in the highveld mole-rat. We exposed mole-rats to a 12L:12D and a DD light cycle at ambient temperatures of 30°C, 25°C and 20°C while locomotor activity and body temperature were measured simultaneously. In addition, we investigated the endogenous rhythms of locomotor activity and body temperature at different ambient temperatures. Mole-rats displayed nocturnal activity at all three ambient temperatures and were most active at 20°C, but least active at 30°C. Body temperature was highest at 30°C and lowest at 20°C, and the daily cycle was highly correlated with locomotor activity. We show that the mole-rats have endogenous rhythms for both locomotor activity and body temperature. However, the endogenous body temperature rhythm appears to be less robust compared to the locomotor activity rhythm. Female mole-rats appear to be more sensitive to temperature changes than males, increased heterothermy is evident at lower ambient temperatures, whilst males show smaller variation in their body temperatures with changing ambient temperatures. Mole-rats may rely more heavily on behavioural thermoregulation as it is more energy efficient in an already challenging environment.

## Introduction

Daily fluctuations in biological functions is a near universal property of living organisms. In rodents, circadian rhythms such as locomotor activity and body temperatures (T_b_), are regulated by internal clocks [1]. The master circadian clock is located in the suprachiasmatic nucleus (SCN), in the basal hypothalamus, and it coordinates the expression of circadian rhythms in the rest of the body [2,3]. Circadian rhythms are synchronised to the external environment by rhythmic environmental cues [4]. The periodic light-dark cycle is the strongest and most reliable cue to which animals entrain their daily rhythms [1].

Despite circadian variation in body temperature, homeotherms display a distinctive ability for thermoregulation [5]. The maintenance of a high and relatively constant T_b_ is advantageous for a number of enzymes and other chemical reactions that are temperature dependent [6]. Maintaining a high T_b_ where performance is optimal is energetically costly, and despite the active employment of thermoregulation, many homeothermic animals still experience considerable variation in T_b_ [5]. Angellitta and colleagues have proposed that varying microclimates impose a selective pressure on the thermosensitivity of performance. This can lead to adaptation of a population by either evolution of thermoregulation (employment of a combination of behaviour, physiology and morphology to regulate T_b_ near the optimum), or evolution of thermosensitivity (ability to function over a wide range of temperatures) [5].

African mole-rats of the family Bathyergidae are endemic to sub-Saharan Africa [7] and all species are subterranean. They rarely venture aboveground and are therefore not frequently exposed to light. Mole-rats are well adapted to their lightless environment; anatomically they possess a regressed visual system [8] and the retention of only basic visual abilities have been shown with behavioural testing [9]. However, the mole-rat circadian system remains intact, and compares anatomically in size to the circadian structures in the brains of other rodents [10–12]. Light also reaches the circadian clock in the SCN, as illustrated by expression of Fos protein in response to light stimulation [13,14]. Furthermore, many mole-rat species can entrain their locomotor activity patterns to light cycles and also display endogenous rhythms of locomotor activity in the absence of light cues [15–18]. Some mole-rat species have also been shown to display daily body temperature rhythms [19], while others do not [20].

The microclimate of the subterranean niche is relatively stable, and temperature ranges within the burrows are fairly muted compared to those on the surface [21,22]. Poor ventilation in the burrow systems restrict gas and heat exchange, exposing the inhabitants to a hypercapnic and hypoxic environment with high humidity levels [23]. The high humidity levels in the burrows prevents mole-rats from employing typical thermoregulatory mechanisms such as convection and evaporative cooling [24]. Mole-rats display a number of physiological adaptations to their habitat, including low resting metabolic rates (RMR’s) and body temperatures (T_b_) [7]. The low RMR’s may contribute to the maintenance of physiological gas partial pressures in mole-rats [25], and can also be responsible for the lower T_b_’s. These attributes, in part, may assist in limiting the risk of overheating in burrows [26].

Bathyergid mole-rats in general show large variation in thermoregulatory abilities. Naked mole-rats (*Heterocephalus glaber*) are considered poikilothermic and at ambient temperatures (T_a_) below 25°C their body temperature may fall more than 5°C [27]. Mashona mole-rats (*Fukomys darling*) have also been shown to show greater heterothermy at lower T_a_’s, and T_b_ can reduce significantly at T_a_ below 25°C, while this appears not to be the case for the highveld mole-rat [28,29]. Social mole-rat species employ behavioural thermoregulation to regulate body temperatures; they select deeper, warmer areas of the burrows for resting [30] and also huddle together to conserve heat [31,32].

This study aimed firstly to determine how the locomotor activity and T_b_ of the highveld mole-rat (*Cryptomys hottentotus pretoriae)* changes over a range of ambient temperatures (T_a_) that they are likely to encounter in their burrows. It has been suggested that the highveld mole-rat is more heterothermic, however, the activity analysis of this study was not very comprehensive [29]. We anticipated that the average T_b_ would remain relatively stable over the range of T_a_’s, but that locomotor activity and the range of T_b_ variation would increase at the lower T_a_‘s. We investigated this by recording T_b_ and locomotor activity over a range of T_a’_s and a 12L:12D light cycle. Secondly we examined the endogenous rhythms of both locomotor activity and T_b_ to determine whether the animals exhibit an endogenous rhythm for T_b_ and how it compares with the activity rhythm, and furthermore whether ambient temperature influences the period of these rhythms. We expected to find that T_b_ rhythms would be less robust than that for locomotor activity, but would show similar period lengths than the endogenous rhythms for locomotor activity.

## Material and Methods

### Ethical statement

All experimental procedures were carried out in accordance with the recommendations in the Guide for the Care and Use of Laboratory Animals of the National Institutes of Health [33]. The protocol was approved by the Ethics Committee of the University of Pretoria–clearance number EC073-13. All surgery was performed under Isofluorane by a registered veterinarian, and all efforts were made to minimize suffering.

### Capture and housing

Sixteen non-reproductive highveld mole-rats were captured in Centurion, Gauteng (25°48’14”S; 28°09’44”E) in South Africa during April/May 2014 using modified Hickman live traps [34] baited with sweet potato. Ten females (mean body mass = 114.50±29.49g) and six males (mean body mass = 132.58±38.72g), originating from 6 colonies, were investigated in this study. One male died (reason unknown) after the first temperature cycle, and was therefore excluded from the analysis.

The animals were transported to the department of Zoology and Entomology, University of Pretoria, Pretoria and housed individually in plastic containers (60x30x30cm) in a light (light phase = 330 lux at floor level (Major tech m940 light meter), dark phase = 0 lux) and temperature controlled room. Containers were lined with wood shavings and each mole-rat was supplied with a nest box and paper tissue for nesting material. The mole-rats were fed an *ad libitum* diet of sweet potato, which was replaced each day at random times. Bennett and Jarvis (1995) investigated the digestibility and nutritional value of sweet potatoes, and found it to provide animals with adequate nutrition. The well-being of the animals was monitored each day during feeding time. The animals were allowed to acclimatize for a week before commencement of the experimental procedures.

### Experimental procedures

Core body temperature was measured with calibrated temperature loggers/iButtons (DS1922L iButtons, MaximIntegrated Products, Dallas, TX, USA) programmed to log a reading hourly with an accuracy of 0.05°C. The iButtons were covered in surgical wax and were surgically implanted intra-peritonially under isoflourane anaesthesia by a registered veterinarian. The data loggers weigh 3.2g which is below 5% of the body weight of the lightest animal implanted. Animals were pre-medicated with butorphanol for anaesthetic and analgesic purposes at a dose of 2mg/kg given intramuscularly. Anesthesia was induced in animals with 5% Isofluorane using an induction chamber and Isofluorane via a Humphreys ADE circle system machine, and maintained via a face mask on between 2 and 3%. Post-surgery, animals were placed back into their containers and monitored during recovery. All animals were awake and grooming within 15 minutes of being removed from the anesthetic. Animals were monitored closely for 24 hours, where after they were returned to the experimental rooms at the University. The animals resumed normal activity almost immediately, and were allowed to recover from the surgery for two days before commencement of the locomotor activity recordings. Upon completion of the experiment, animals were euthanized with an overdose of halothane, where after the data loggers were extracted and data downloaded.

Locomotor activity was measured using infrared motion detectors (Quest PIR internal passive infrared detector; Elite security products (ESP), Electronic lines, UK) that were fitted onto each plastic container in such a way that the entire floor space of the container was covered. Collective activity measurements were recorded once per minute and captured on a computer situated outside the activity room by the program Vital View (Vital View^TM^, Minimitter Co., Inc., Sunriver, OR, USA; www.minimitter.com). All animals were subjected consecutively to three different ambient temperatures (30°C, 25°C and 20°C) both on an LD light cycle (12L:12D; light from 07:00–19:00) and under constant darkness cycle (DD). The experimental procedure was as follows: 30°C LD for 25 days, 30°C DD for 26 days, 25°C LD for 25 days, 25°C DD for 25 days and lastly 20°C LD for 28 days and 20°C DD for 29 days. All mole-rats were subjected to all the light and temperature cycles. iButtons were also used to measure soil temperatures on the surface (shade and sun), as well as at 5, 15, 25, 35 and 45cm, the iButtons were placed at a nearby site where they could be left undisturbed for 5 days.

### Data analysis

Activity measurements were depicted as double plotted actograms using Actiview Biological Rhythm Analyses 1.2 software (Minimitter Co., Inc., Sunriver, OR, USA; www.minimitter.com), to offer a visual representation of individual activity patterns. As a result of the length of the study, actograms presented in the manuscript are shown separately for each ambient temperature, complete actograms of all animals are presented in the supplementary material (S3 Fig). The data from the first week of each light cycle was excluded from the analysis to exclude potential aftereffects of the previous cycles. Activity counts were summed to hourly values for comparison with the T_b_ readings. For T_b_, the mean, minimum, maximum and variation was calculated for each 24h period during LD. As a measure of variation, the minimum T_b_ was deducted from the maximum T_b_. Since data were not normally distributed, non-parametric statistical tests in SPSS 22 (SPSS Inc., Chicago, Illinois) were used for analysis. We used Generalized Linear Mixed Models to evaluate locomotor activity and mean, minimum, maximum and variation in body temperature. Generalised linear model repeated measures were used with gender and the respective ambient temperatures as fixed factors. Body mass was also controlled for. The period lengths for the locomotor activity and body temperature rhythms during constant conditions were determined using the Chi-square periodogram function within the Clocklab software (ClockLab™; Actimetrics, Evanston, Illinois, USA). The correlation between the lengths of the endogenous rhythms of locomotor activity and Tb were determined with a non-parametric correlation.

## Results

### Soil temperatures

Surface and soil temperatures were measured for 5 days during the same time of year that the mole-rats were trapped. The mean ambient temperatures in full sun was comparable to that 5 cm below ground, although the temperature range in full sun was much more extensive. The mean temperatures increased progressively with depth below the ground, while the standard deviation decreased. Minimums, maximums, means and standard deviations of the surface and soil temperatures are listed in Table 1. See supporting information S1 Fig. Soil temperature, for a graphic representation of the temperatures at the different depths over the 5-day period.

**Table 1:** Mean temperature ranges (°C) from two i-buttons buried at different depths in the soil.

	Min	Max	Mean ± SD
Full sun	6.36	45.17	17.81±10.87
Shade	7.71	24.43	15.07±4.28
5cm	11.63	29.39	17.41±4.02
15cm	14.77	22.90	18.41±2.03
25cm	17.11	20.99	19.39±1.00
35cm	18.21	20.83	19.90±0.65
45cm	19.06	20.99	20.32±0.47

### Daily activity and body temperature under 12L:12D

**Fig 1:**
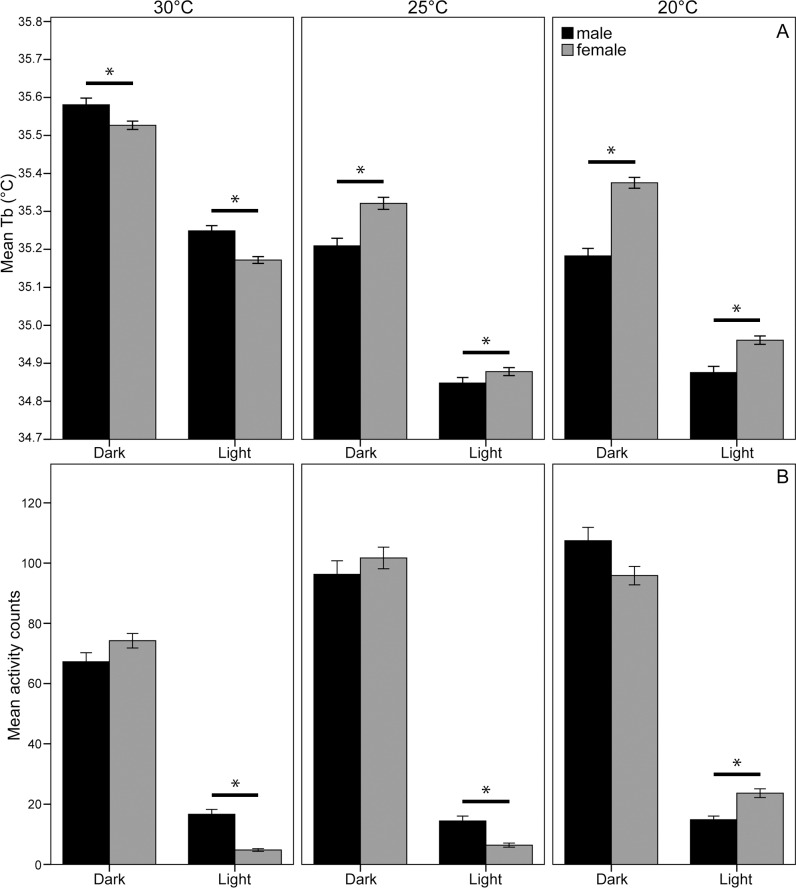
(A) Mean body temperatures for female and male highveld mole-rats during the light and dark periods of the LD cycles at the three ambient temperatures tested. (B) Mean locomotor activity counts for the highveld mole-rats during the light and dark periods of the LD cycles at the three different ambient temperatures tested. Significant differences are indicated with * and error bars are for SE.

**Fig 2:**
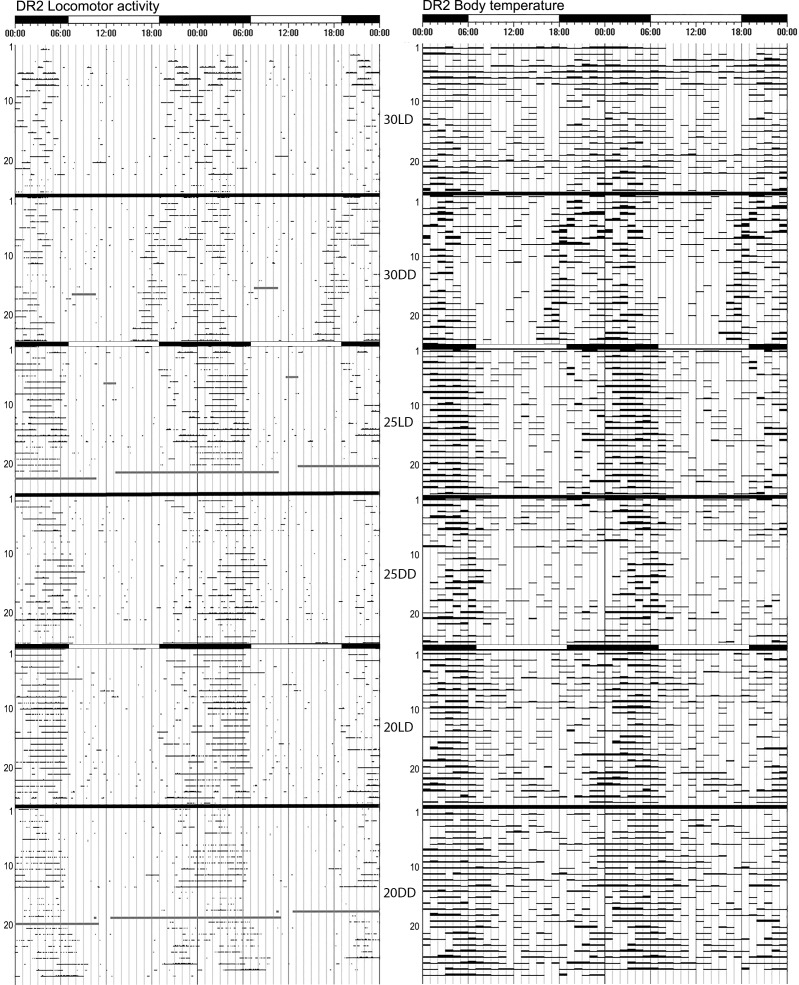
Actograms of the locomotor activity and core T_b_ of mole-rat DR2 at 30°C, 25°C and 20°C,showing good entrainment under LD conditions and clear free-running rhythms under constant darkness. The free-running periods for constant darkness were clearly discernible and were similar for locomotor activity and T_b_. The black and white bars on top of the actograms shows the dark and light phases during the LD cycles, during DD cycles no light is present. The number of days are on the Y-axis, and the experimental conditions are indicated between the two actograms.

**Fig 3:**
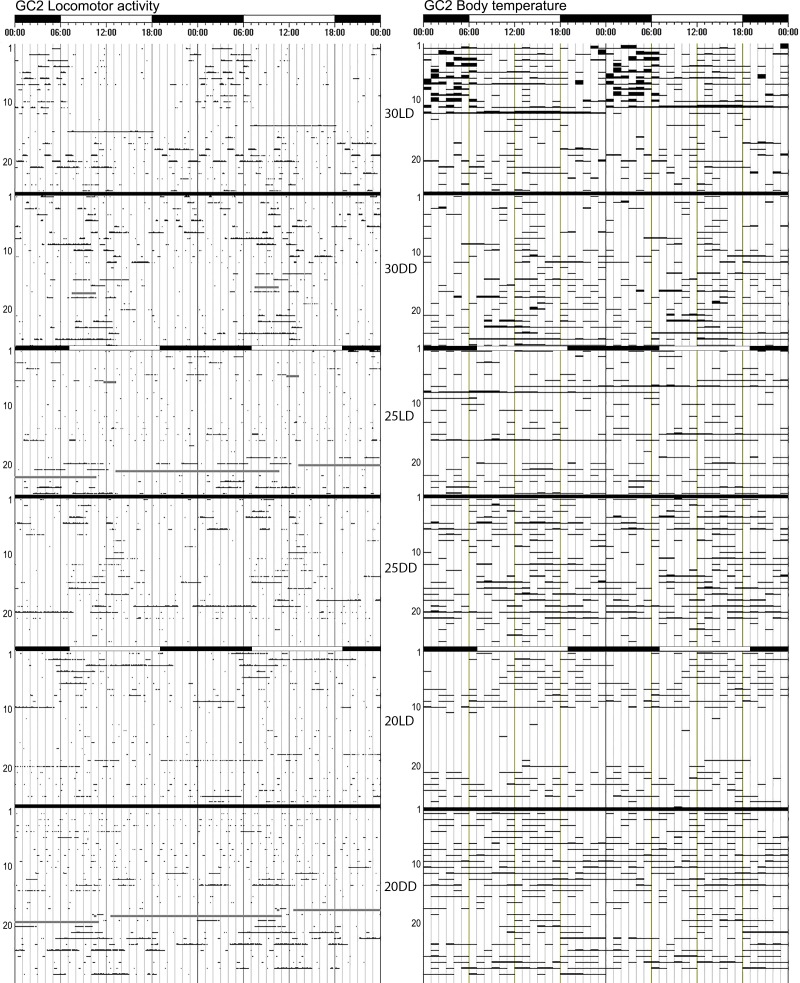
Actograms of the locomotor activity and core T_b_ of mole-rat GC2 at 30°C, 25°C and 20°C, showing weak entrainment under the LD cycles and no obvious free-running rhythm under constant darkness. The black and white bars on top of the actograms shows the dark and light phases during the LD cycles, during DD cycles no light is present. The number of days are on the Y-axis, and the experimental conditions are indicated between the two actograms.

Entrainment was defined as a stable phase angle between the onset or offset of the light phase and the onset or offset of the activity. At an ambient temperature of 30°C, 11 of the 15 animals showed clear entrainment while the remaining 4 animals showed low activity or no entrainment. At ambient temperatures of 25°C and 20°C, 9 animals exhibited good entrainment whereas the remainder of the animals were arrhythmic or had low activity (Figs 1, 2 and 3; Table 2). See supporting information S2 Fig. Daily mean T_b_ and activity, for the 24h T_b_ and activity curves. Complete actograms of all animals are included in the supporting information S3 Fig. Actograms.

**Table 2:** Mean overall activity counts (±SE) and mean overall body temperatures (C°) (±SE) for the light and dark phases of the 12L:12D light cycle for highveld mole-rats at the three ambient temperatures tested, as well as separated between genders. Standard deviations are indicated in brackets below.

Ambient	Light		Dark		Gender	Light		Dark	
Temperature	Activity	Tb	Activity	Tb		Activity	Tb	Activity	Tb
20°C	20.7±1.05 (65.87)	34.93±0.01(0.57)	99.72±2.51 (158.11)	35.31±0.01 (0.74)	♂	14.83±1.19(43.15)	34.89±0.03 (0.59)	107.44±4.42 (160.61)	35.2±0.03 (0.72)
					♀	23.64±1.65(74.52)	35.02±0.02(0.57)	95.94±3.05(156.73)	35.44±0.02(0.74)
25°C	9.07±0.7 (39.68)	34.87±0.01(0.5)	99.9±2.83 (159.87)	35.28±0.01 (0.73)	♂	14.41±1.6 (52.69)	34.86±0.03 (0.5)	96.29±4.52 (148.18)	35.22±0.03 (0.67)
					♀	6.37±0.67 (30.76)	35.16±0.02 (0.5)	102.18±3.6 (165.48)	35.38±0.02 (0.76)
30°C	8.73±0.62 (41.54)	35.2±0.01 (0.51)	71.93±1.89 (126.96)	35.54±0.01 (0.63)	♂	16.64±1.59(61.42)	35.25±0.03 (0.53)	67.28±2.97 (114.97)	35.58±0.03 (0.68)
					♀	4.77±0.47 (25.61)	35.16±0.02 (0.5)	74.26±2.45 (132.5)	35.52±0.02 (0.6)

Highveld mole-rats were significantly more active during the dark phase of the 12L:12D light cycle (F = 581.23, df_1_ = 1, df_2_ = 23,316, p<0.001; Fig 1). Mean body temperatures of the mole-rats were also higher during the dark phase compared to the light phase (F = 2,126, df_1_ = 1, df_2_ = 23,751, p<0.001; Fig 1). The mean overall activity was not different between males and females (F = 3.135, df_1_ = 1, df_2_ = 23.316, p = 0.077), activity of males and females was similar during the dark phases of all the ambient temperatures, however, males showed more activity than females during the light phase at 30°C (F = 6.96, df_1_ = 1, df_2_ = 12.884, p = 0.008) and 25°C (F = 19.72, df_1_ = 1, df_2_ = 12.884, p<0.05), whereas females were more active during the light phase at 20°C (F = 4.49, df_1_ = 1, df_2_ = 12.884, p = 0.034), Fig 1). Females had higher T_b_ than males overall (F = 8.2, df_1_ = 1, df_2_ = 23,75127.588, p = 0.004). The mean T_b_ of females was higher compared to males for both light and dark phases at 20°C (light: F = 17.02, df_1_ = 1, df_2_ = 23,751, p<0.001; dark: F = 55.48, df_1_ = 1, df_2_ = 23,751 p<0.001) and 25°C (light: F = 4.8, df_1_ = 1, df_2_ = 23,751, p = 0.028; dark: F = 21.75, df_1_ = 1, df_2_ = 23,751, p<0.001), but at 30°C, males had higher Tb compared to the females for both the light (F = 7.32, df_1_ = 1, df_2_ = 23,751, p = 0.007) and the dark phase (F = 3.86, df_1_ = 1, df_2_ = 23,751, p = 0.05; Fig 1; Table 2).

Mean activity counts of highveld mole-rats differed significantly between all ambient temperatures, they were most active at 20°C and least active at 30°C (F = 89.19, df_1_ = 2, df_2_ = 23,316, p<0.001; Fig 1). In both genders, ambient temperature significantly affected mean activity during both the dark (♀: F = 26.74, df_1_ = 2, df_2_ = 23,316, p<0.001; ♂: F = 17.87, df_1_ = 2, df_2_ = 23,316, p<0.001) and light phases (♀: F = 94.1, df_1_ = 2, df_2_ = 23,316, p<0.001; ♂: F = 4.46, df_1_ = 2, df_2_ = 23,316, p = 0.012). A significant difference was apparent in the mean T_b_ between all three ambient temperatures in females (F = 119.66, df_1_ = 2, df_2_ = 23,751, p<0.001) where the highest T_b_ is observed at 30°C, and the lowest at 25°C. In males, ambient temperature influenced T_b_ (F = 324.04, df_1_ = 2, df_2_ = 23,751, p<0.001), with T_b_ at 30°C significantly higher than at 25°C (p<0.001) and 20°C (p<0.001). T_b_ at 25°C and 20°C did not differ. Males also show the highest T_b_ at 30°C, but their body temperature was lowest at 20°C (Fig 1; Table 2).

### Variation in body temperature

**Fig 4:**
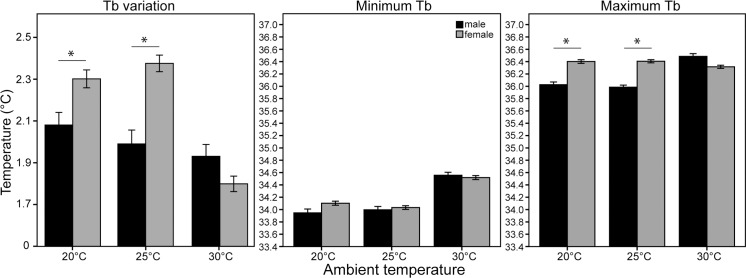
Differences between male and female highveld mole-rats at the three different ambient temperatures tested, for variation between maximum and minimum T_b_, minimum and maximum body temperatures (T_b_’s) respectively. Significant gender differences are indicated with * and error bars are for SE.

Ambient temperature significantly affects the variation between T_b_ max and T_b_ min (F = 49.3, df_1_ = 2, df_2_ = 1,138, p<0.001). The amount of variation between 20°C and 25°C is similar (p = 0.893), but the variation differs significantly between 30°C and both 20°C and 25°C (p<0.001 for both comparisons). Likewise, the minimum T_b_ differed significantly at the different temperatures (F = 134.57, df_1_ = 2, df_2_ = 1,158, p<0.001), although the minimum T_b_ was comparable at 20°C and 25°C (p = 0.123), at 30°C it was higher than both 20°C and 25°C (p<0.001 for both comparisons). Maximum T_b_ also differed significantly at the various ambient temperatures (F = 13.76, df_1_ = 2, df_2_ = 1,158, p<0.001). Again 20°C and 25°C did not differ (p = 0.276), but the maximum T_b_ at 30°C was significantly higher than at the other two temperatures (both p<0.001, Fig 4).

Female highveld mole-rats showed significantly more variation in their body temperatures compared to the males at 20°C (F = 8.2, df_1_ = 1, df_2_ = 1,138, p = 0.004) and 25°C (F = 27.9, df_1_ = 1, df_2_ = 1,138, p<0.001) but not at 30°C (F = 1.3, df_1_ = 1, df_2_ = 1,138, p = 0.254). No gender difference was evident in the minimum T_b_‘s (F = 0.46, df_1_ = 1, df_2_ = 1,158, p = 0.498), but females displayed significantly higher maximum T_b_‘s compared to the males (F = 59.44, df_1_ = 1, df_2_ = 1,158, p<0.001; Fig 4).

### Circadian cycles in constant conditions

**Fig 5:**
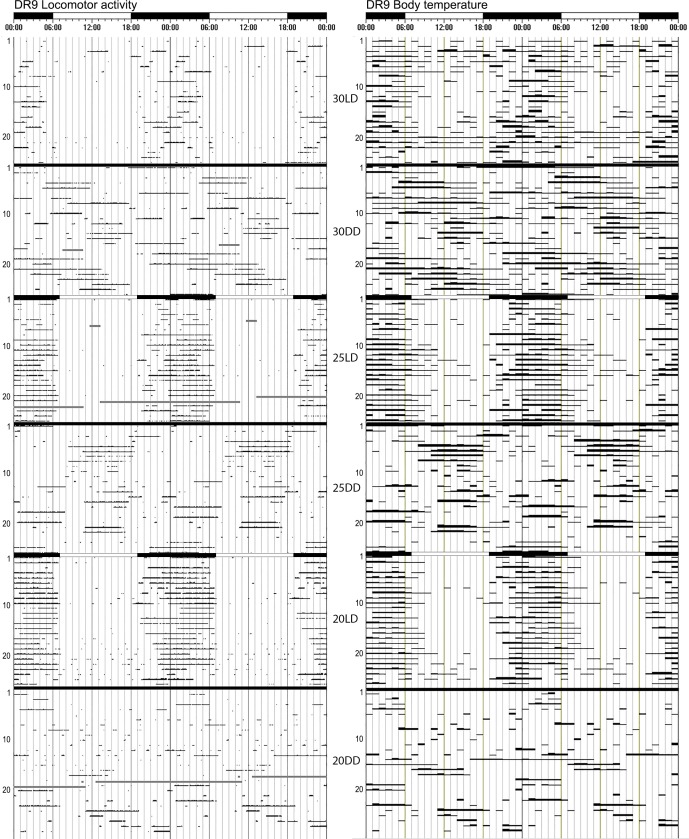
(A) Actograms of the locomotor activity and core T_b_ of mole-rat DR9 showing good entrainment under the LD cycles and very long and ill-defined free-running rhythms under constant darkness. The black and white bars on top of the actograms shows the dark and light phases during the LD cycles, during DD cycles no light is present. The number of days are on the Y-axis, and the experimental conditions are indicated between the two actograms.

A free-running rhythm for locomotor activity could be calculated for all 15 mole-rats at all three ambient temperatures, albeit rather weak for some. The mean period length (Ƭ) of the locomotor activity rhythms was slightly longer than 24h at all three temperatures (20°C: 24h14 ± 0h57; 25°C: 24h09 ± 0h30; 30°C: 24h20 ± 0h53). Overall, there was no significant difference in the Ƭ of the locomotor activity rhythm at different ambient temperatures (F = 0.87, df_1_ = 2, df_2_ = 39, p = 0.426) (Figs 2, 3 and 5).

The length of the free-running rhythm of the body temperature could be determined in 12 animals at 20°C and in 14 animals each at 25°C and 30°C. The mean period length of body temperature endogenous rhythms was also longer than 24h for all three ambient temperatures (20°C: 24h10 ± 0h59; 25°C: 24h23 ± 0h46; 30°C: 24h10 ± 0h32). The Ƭ of the T_b_ circadian rhythms did not differ at the various ambient temperatures (F = 0.39, df_1_ = 2, df_2_ = 28, p = 0.68) (Figs 2, 3 and 5). The period lengths of the locomotor activity and Tb rhythms were highly correlated (r = 0.751, p<0.001).

The period length of the circadian locomotor activity rhythm was significantly longer in males than in females (F = 10.53, df_1_ = 1, df_2_ = 33, p = 0.003). This turned out to be as a result of a single male with an exceptionally long circadian period (Fig 5). If this animal is removed from the analysis, there is no difference between males and females (F = 0.63, df_1_ = 1, df_2_ = 30, p = 0.435). Upon the removal of removal of the animal with the long activity period, the Ƭ of the T_b_ circadian rhythm was similar in males compared to females (F = 0.45, df_1_ = 1, df_2_ = 26, p = 0.507).

## Discussion

### Daily locomotor activity and body temperature

Time of activity is generally assumed to have an adaptive advantage that is species specific [35]. Since mole-rats are subterranean and come above ground very infrequently, light is likely to be of lesser importance in the determination of locomotor activity times and the adaptive significance of robust activity rhythms may be less obvious. However, many mole-rat species investigated do show distinct daily locomotor activity rhythms under specific lighting regimes [15,17,18].

In the current study, all highveld mole-rats displayed more activity during the dark phase of the light cycles and this was true for all ambient temperatures tested. This result is consistent with what has been previously reported for this species [15]. Most other mole-rat species also exhibit nocturnal activity when exposed to a 12L:12D light cycle [15,17,18,36]. A nocturnal activity pattern was observed in both males and females. Although no gender difference was observed in the overall activity counts, the temporal distribution of activity was different for males and females. Activity levels were similar for males and females during the dark phases of all ambient temperatures, but males were more active during the light phases of the higher ambient temperatures, while females were more active during the light phase of the coolest ambient temperature. Sexual dimorphism in locomotor activity is not common in rodents; several other species investigated show no gender differences in locomotor activity patterns, both with and without running wheels [37]. However, some cases of gender differences have been reported, male gerbils show running wheel activity during the day, whereas females do not, but females are overall more active [38], and female Syrian hamsters show higher levels of activity than the males [39].

Our data revealed that locomotor activity and body temperatures (T_b_) of the highveld mole-rats are highly correlated, such that the T_b_ rises as activity increases and that both parameters reach a peak at about the same time. Peak values for both activity and body temperature occur at night for nocturnal animals and activity and T_b_ peaks have been reported as being synchronous in rats [1]. Lower body temperatures may occur during inactive periods, for example during sleep [40], but the lowest T_b_’s for the mole-rats occurred mostly immediately after high levels of activity. Female mole-rats displayed higher T_b_ compared to males at the lower ambient temperatures, while males had higher T_b_ at the highest ambient temperature. As far as we are aware, there are no gender differences in tasks performed in the colony that could account for the difference in T_b_, nor are there any known physiological differences between males and females. However, no dedicated studies have been performed to look at this phenomenon, and this may be something that should be explored.

As can be seen from our soil temperature results, mole-rats are not exposed to extensive ambient temperature changes in their natural habitat. Hence they may be more sensitive to small temperature changes occurring within the burrow system. Ambient temperature played a significant role in the amount of locomotor activity exhibited by highveld mole-rats. Animals were most active at 20°C, and became less active as the ambient temperature increased. Another mole-rat species, the Damaraland mole-rat has also been found to display reduced levels of activity at 30°C when subjected to a similar temperature regime [36]. A reduction in activity levels when the ambient temperature approaches the thermoneutral zone (TNZ) has also been reported for other species such as laboratory mice [41]. In social mole-rat species, the TNZ’s typically range between 28–36°C [6,29,32,42,43]. The TNZ of the highveld mole-rat is around 30–32°C [44], thus 30°C falls within this zone.

Within the TNZ of an animal, the basal rate of heat production is equivalent to the rate of heat loss to the environment, therefore no energy is expended to maintain body temperature. In general, animals appear to be comfortable in this zone, and have been shown to prefer ambient temperatures that overlap with this zone for low intensity activities such as resting and sleeping [41]. Small and medium sized mole-rats have been reported to choose ambient temperatures close to 30°C for resting, while larger mole-rat species preferred slightly cooler ambient temperatures [45]. Reduced activity at higher ambient temperatures may be a mechanism employed to avoid hyperthermia [46] however, temperatures close to the TNZ of animals may simply be energetically less demanding for the animals and high levels of activity are not needed to maintain body temperatures.

At ambient temperatures below the TNZ, animals would need to generate more heat to maintain a constant T_b_. This can be achieved using behavioural thermoregulation in the form of increased locomotor activity or by huddling to conserve heat [25]. Highveld mole-rats are social animals, and in their natural habitat, they may huddle together in communal nests to conserve body heat. However, in the present study mole-rats were housed individually in order to record individual activity, removing the option of huddling. Hence the increase in locomotor activity at lower T_a_’s observed here may be induced by the experimental conditions rather than what would occur in nature.

### Variation in body temperature

At ambient temperatures overlapping with the TNZ of the animals, the minimum and maximum Tb of all animals were higher than at ambient temperatures below the TNZ. The mole-rats are likely not exposed to temperatures in their TNZ for extended periods of time in their burrows. Thus at higher ambient temperatures, a theoretical optimal T_b_ may be a relative concept. It may be a trade-off between the energetic costs of reducing Tb by metabolic means or maintaining the Tb at the lowest level behavioural thermoregulation allows for.

Although highveld mole-rats are classified as homeothermic, they still experience temporal variations in body temperature. Our data indicate that highveld mole-rats show increased daily T_b_ variations with decreased ambient temperatures. Although the males showed a difference between the daily maximum and minimum T_b_, this difference is more pronounced in females, particularly at the lower temperatures. These results may indicate that female highveld mole-rats are more sensitive to changes in ambient temperature than the males. Body temperatures of free-ranging Damaraland mole-rats were also found to vary considerably more during winter compared to summer [6], and the higher variability at cooler ambient temperatures appears to be consistent with the results of the females in our study.

In congruence with the above, female highveld mole-rats showed significantly more variation in their body temperatures compared to the males. Interestingly, there were no difference in the minimum T_b_’s, but females displayed significantly higher maximum T_b_’s. Females seem to maintain their T_b_ above a certain minimum, and are able to let their T_b_ vary over a greater range of temperatures than the males. A gender difference was also apparent in Damaraland mole-rats, but there the minimum T_b_’s differed between the genders, with females showing higher minimum T_b_’s [6]. Adjustments to thermoregulatory responses appear to be species specific in mole-rats although females seem to be consistently warmer than males.

### Circadian rhythms in locomotor activity and body temperature

Highveld mole-rats showed endogenous rhythms of both locomotor activity and body temperature, but the body temperature rhythms were detected in a smaller number of animals. The length of these endogenous rhythms were slightly longer than 24 hours, the majority of the rhythms had lengths close to 24h, with one mole-rat showing very long Ƭ (tau) for its rhythms (almost 28 h). This long Ƭ may potentially be as a result of a genetic mutation, several mutations in other rodent species have been shown to result in either long or short periods for circadian rhythms [47,48]. The length of circadian rhythms may not be critical for the survival of strictly subterranean mammals on a daily basis, however it may affect seasonal behaviours.

Locomotor activity rhythms have previously been described for highveld mole-rats [15], however, this is the first report of body temperature rhythms for this species. Circadian rhythms of body temperature have been observed in naked mole-rats [19] and blind mole-rats [49], but Cape mole-rats showed a lack of an obvious endogenous rhythms for body temperature [20]. Locomotor activity rhythms and T_b_ rhythms of the highveld mole-rats were highly correlated. Since body temperature rises with increased locomotor activity, it may be argued that the body temperature rhythm is merely an effect of the locomotor activity expressed. However, the body temperature rhythm lengths are not exactly equal to those of the locomotor activity rhythms, also not all animals that exhibit discernible locomotor activity rhythms, express body temperature rhythms, suggesting an independent mechanism responsible for the body temperature rhythm. Body temperature rhythms appear to be less stable than the activity rhythms, although both the locomotor activity and body temperature are more arrhythmic in the TNZ.

A large number of other species, both nocturnal and diurnal, terrestrial and subterranean, also exhibit these two rhythms to be closely matched in length [19,49–51]. Neither the tau for locomotor activity nor the T_b_ of highveld mole-rats varied with the different ambient temperatures. This was to be expected since circadian rhythms are temperature compensated [52].

### Concluding remarks

Highveld mole-rats could entrain their locomotor activity and body temperature to light cycles, and exhibited circadian rhythms in the absence of external cues. Entrainment and endogenous rhythms of locomotor activity have previously been shown for this species, but this is the first record for body temperature rhythms for highveld mole-rats. The endogenous circadian rhythms for locomotor activity and body temperature were found to be highly correlated. Both the locomotor activity and body temperatures of highveld mole-rats are influenced by ambient temperature, and these two parameters show an inverse relationship. Increased heterothermy is evident at lower ambient temperatures in the females, but not the males. It therefore appears that female highveld mole-rats are more sensitive to changes in ambient temperature than males, but It is not clear why this gender difference in thermoregulation exists. Thermoregulatory abilities of the highveld mole-rats are comparable to what has been reported in other mole-rat species. The relatively stable and moderate environmental conditions in the underground niche may have reduced the selective pressure for physiological thermoregulation in mole-rats. Behavioural thermoregulation may be sufficient and more energy efficient in an already challenging environment.

## Supporting Information

S1 Fig Soil temperature. Ambient temperatures (°C) in full sun, shade and at different soil depths over five consecutive days.
(TIF)

S2 Fig Daily mean T_b_ and activity. Mean body temperature (±SE) and mean activity counts (±SE) for female and male highveld mole-rats over the 24h day at each of the three ambient temperatures tested. Black bars indicate the dark phase of the light cycle and white bars indicate the light phase.
(TIF)

S3 Fig Actograms. Complete actograms for the duration of the experimental procedure are presented for all animals. The black and white bars on top of the actograms shows the dark and light phases during the LD cycles, during DD cycles no light is present. The number of days are on the Y-axis.
(ZIP)
