# Supplementary figures and images for: Locomotor Activity and Body Temperature Patterns over a Temperature Gradient in the Highveld Mole-Rat (Cryptomys hottentotus pretoriae)
Source: PLoS One. 2017 Jan 10;12(1):e0169644. doi: 10.1371/journal.pone.0169644 (PMC5224861; doi:10.1371/journal.pone.0169644)

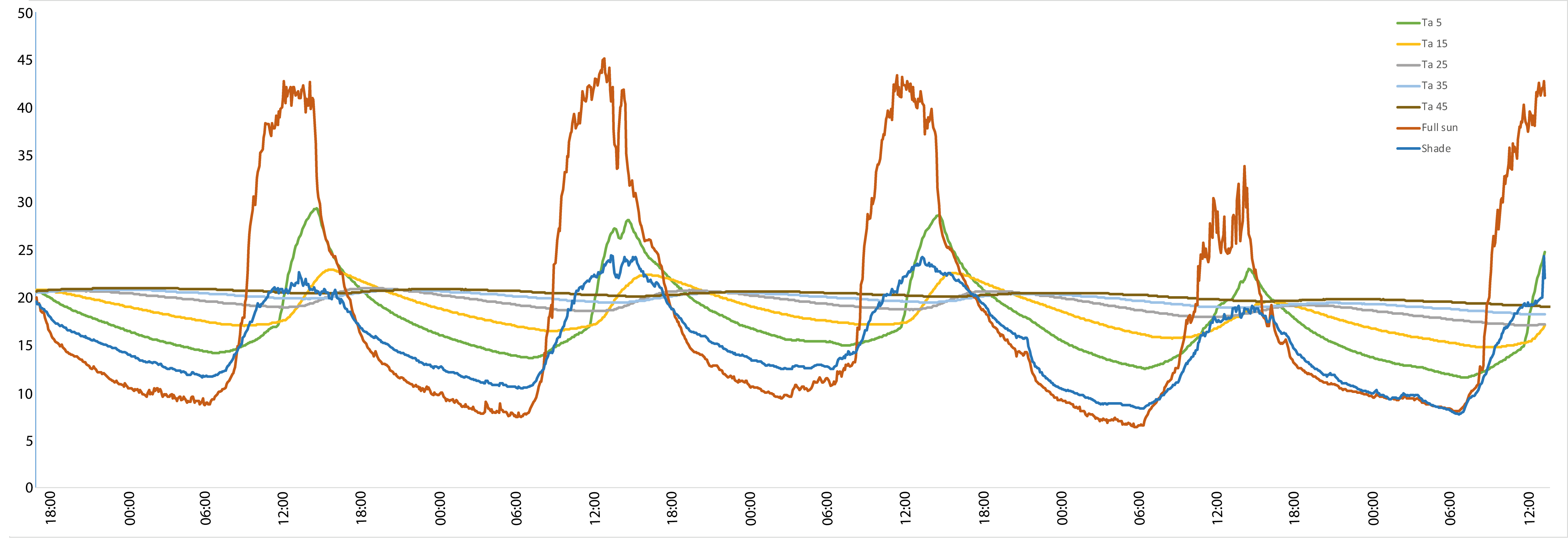

Supplement: S1 Fig — Ambient temperatures (°C) in full sun, shade and at different soil depths over five consecutive days. (TIF) [file pone.0169644.s001.tif]

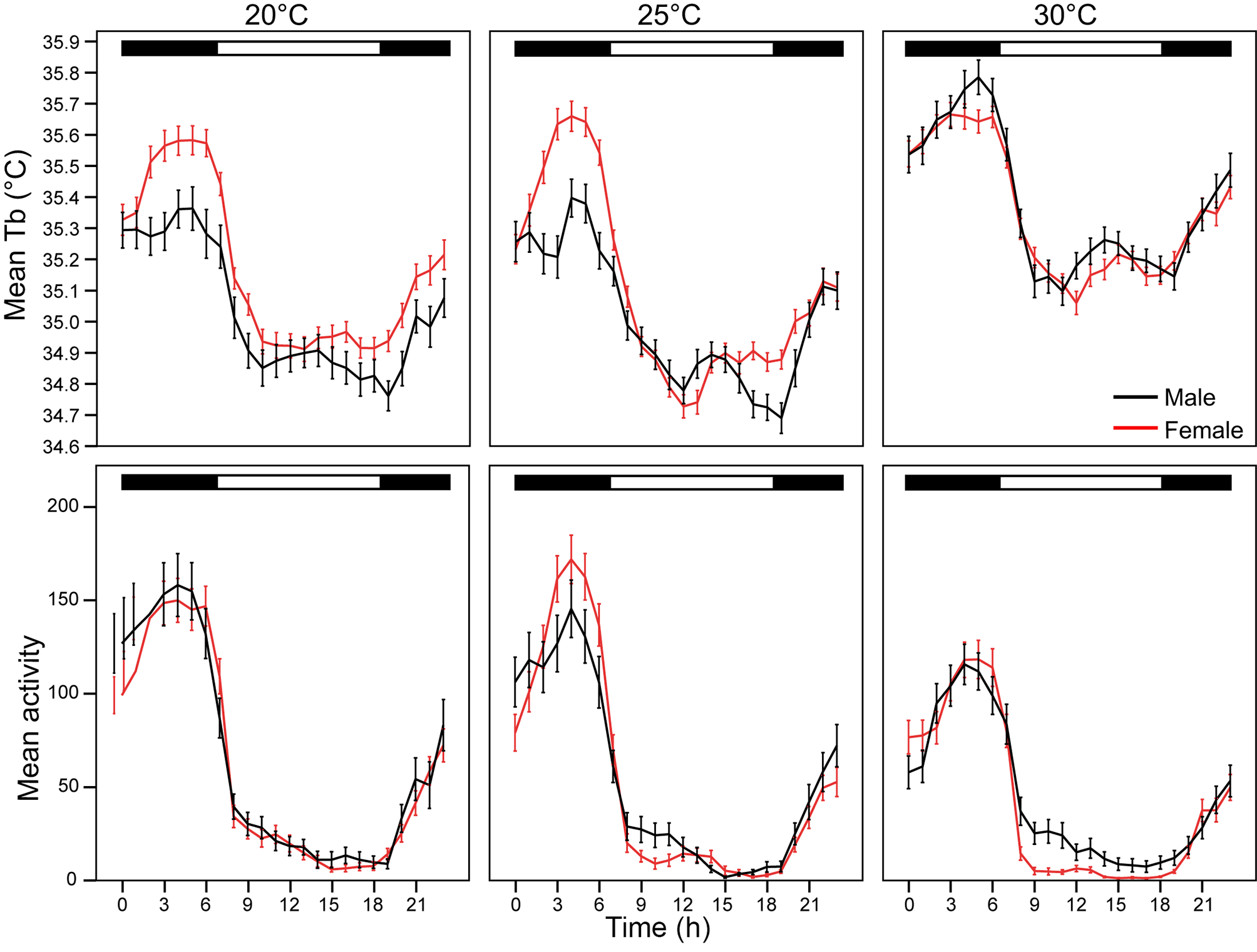

Supplement: S2 Fig — Mean body temperature (±SE) and mean activity counts (±SE) for female and male highveld mole-rats over the 24h day at each of the three ambient temperatures tested. Black bars indicate the dark phase of the light cycle and white bars indicate the light phase. (TIF) [file pone.0169644.s002.tif]

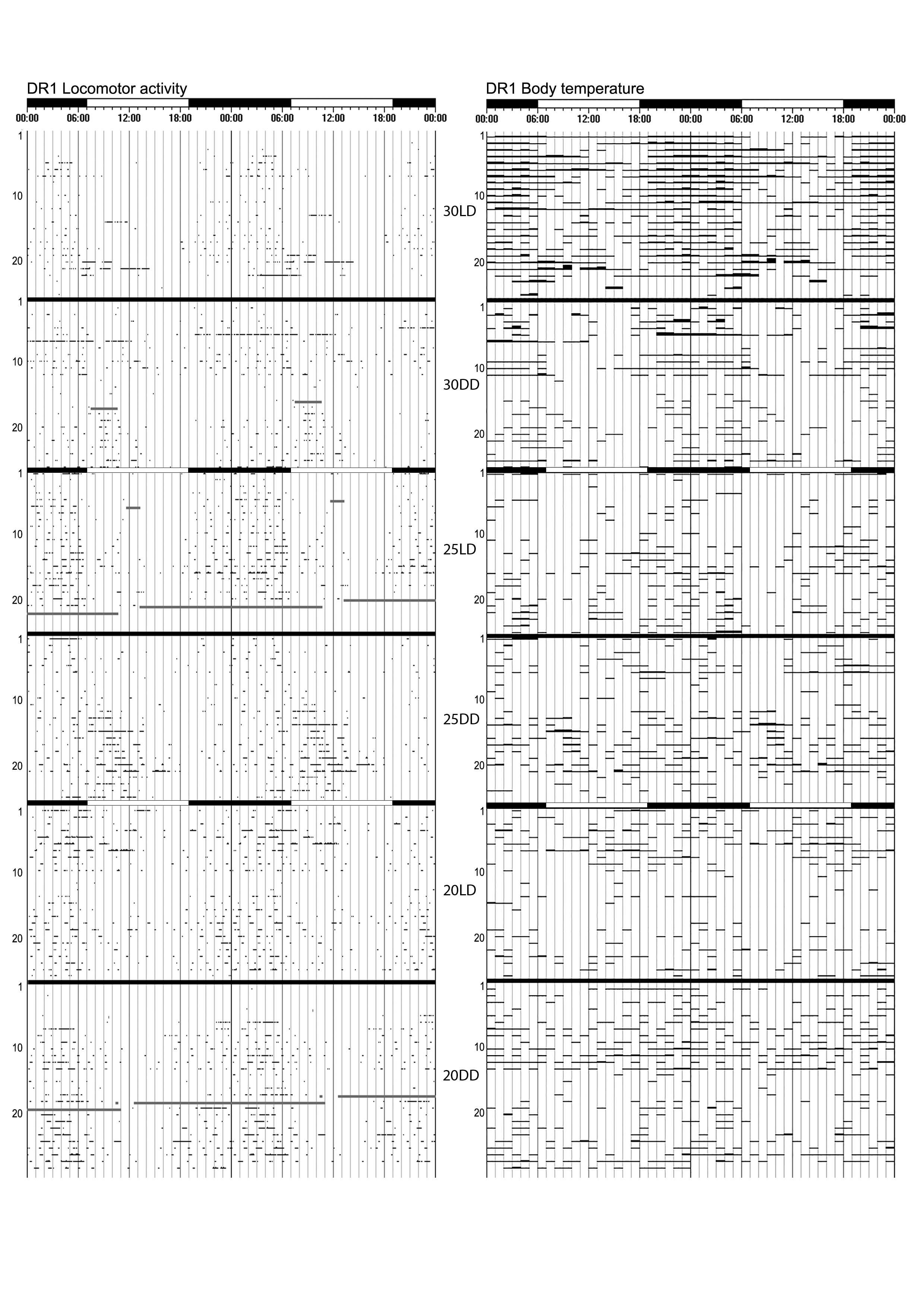

Supplement: S3 Fig — Complete actograms for the duration of the experimental procedure are presented for all animals. The black and white bars on top of the actograms shows the dark and light phases during the LD cycles, during DD cycles no light is present. The number of days are on the Y-axis. (ZIP) [file pone.0169644.s003.zip › Supplementary material/S3 DR1 activity and Tb.tif]

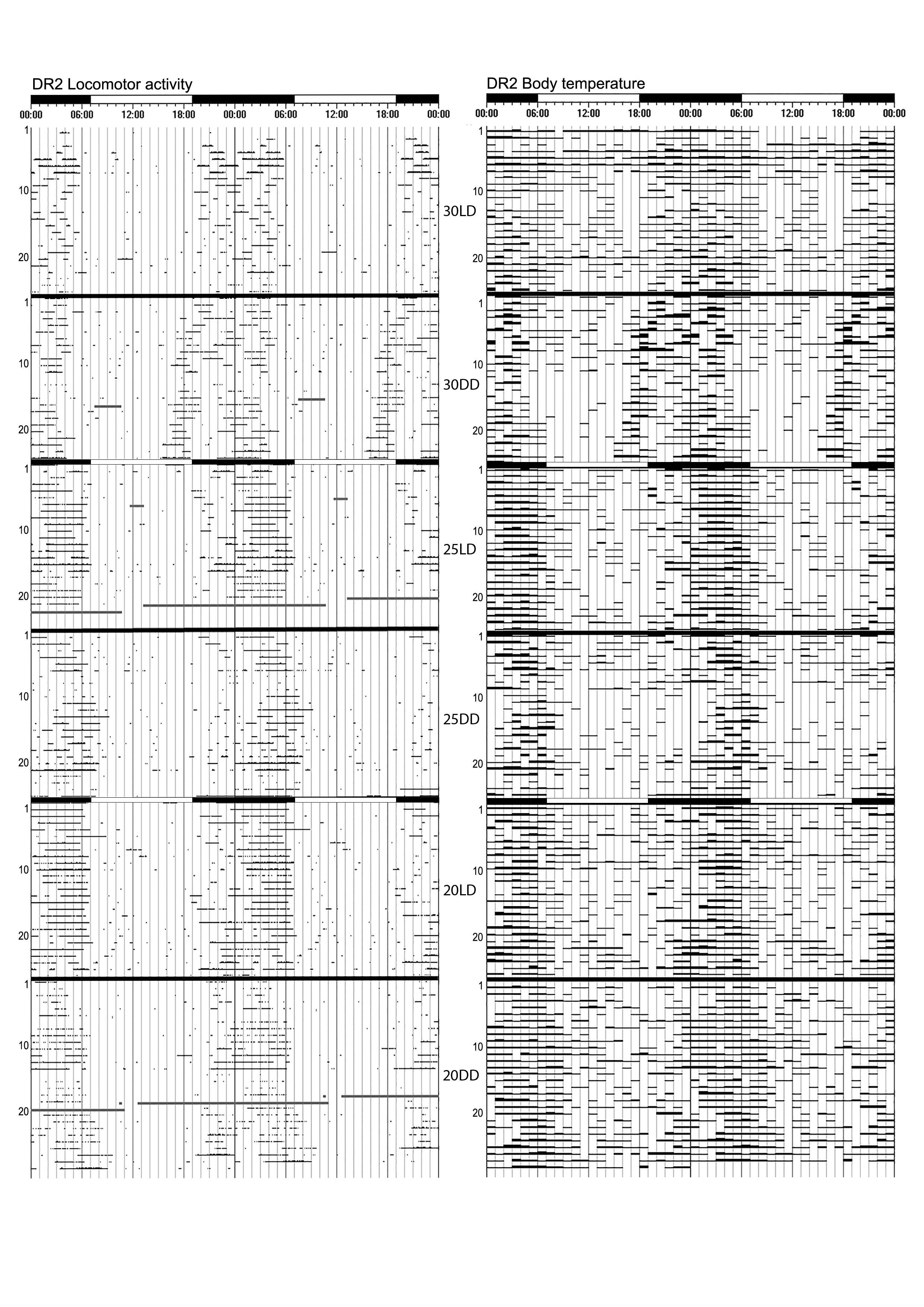

Supplement: S3 Fig — Complete actograms for the duration of the experimental procedure are presented for all animals. The black and white bars on top of the actograms shows the dark and light phases during the LD cycles, during DD cycles no light is present. The number of days are on the Y-axis. (ZIP) [file pone.0169644.s003.zip › Supplementary material/S3 DR2 act & Tb.tif]

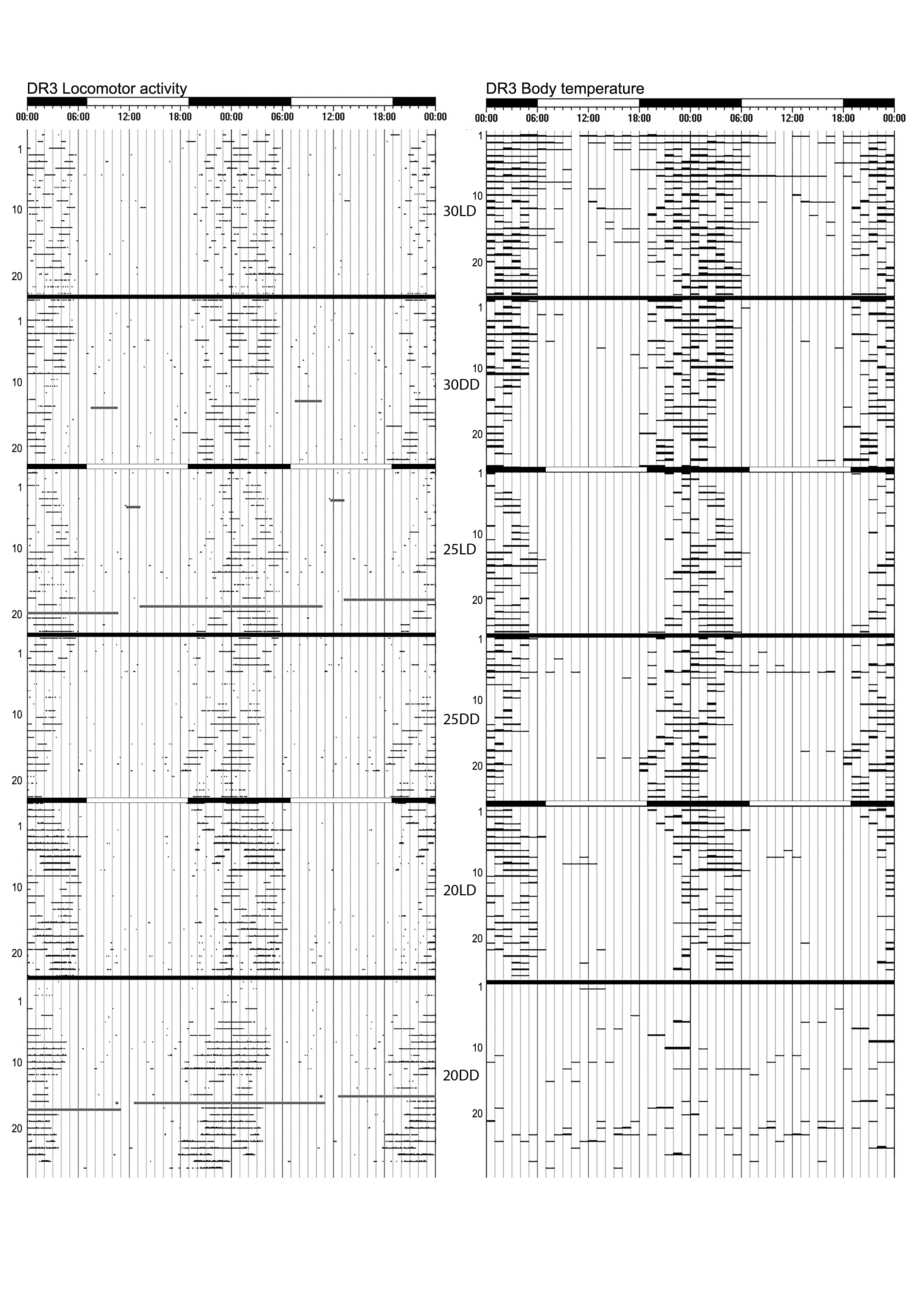

Supplement: S3 Fig — Complete actograms for the duration of the experimental procedure are presented for all animals. The black and white bars on top of the actograms shows the dark and light phases during the LD cycles, during DD cycles no light is present. The number of days are on the Y-axis. (ZIP) [file pone.0169644.s003.zip › Supplementary material/S3 DR3 activity and Tb.tif]

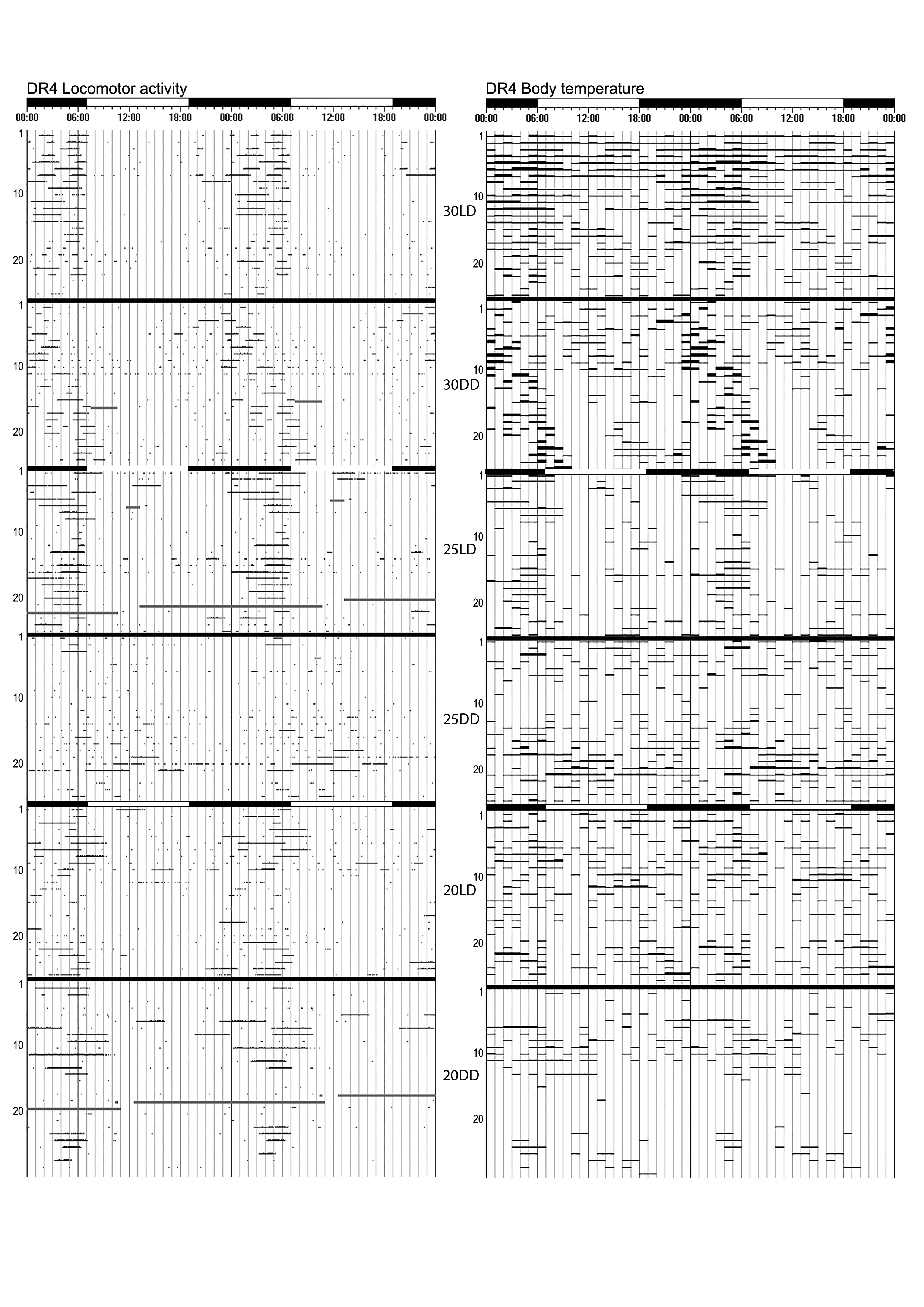

Supplement: S3 Fig — Complete actograms for the duration of the experimental procedure are presented for all animals. The black and white bars on top of the actograms shows the dark and light phases during the LD cycles, during DD cycles no light is present. The number of days are on the Y-axis. (ZIP) [file pone.0169644.s003.zip › Supplementary material/S3 DR4 activity and Tb.tif]

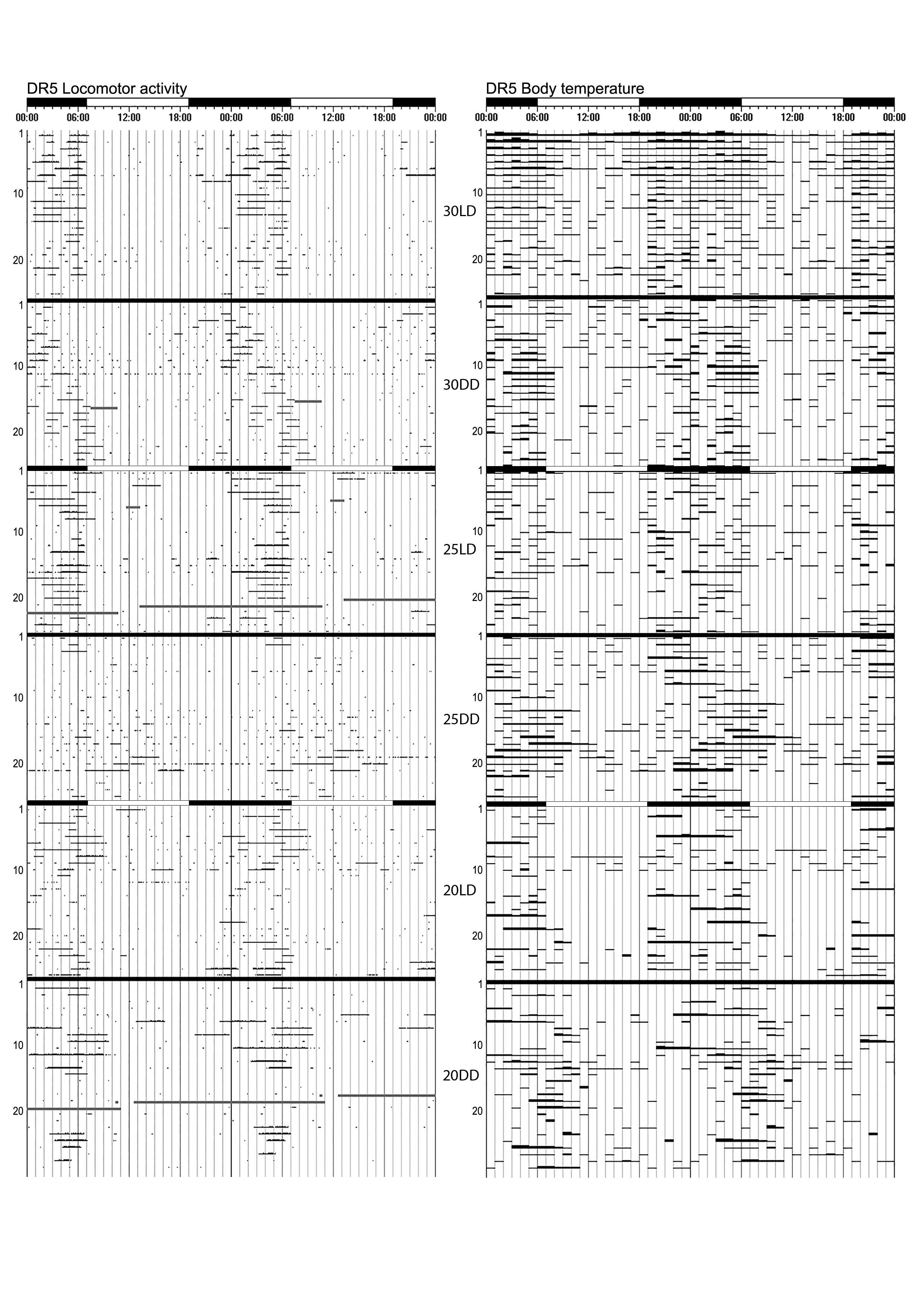

Supplement: S3 Fig — Complete actograms for the duration of the experimental procedure are presented for all animals. The black and white bars on top of the actograms shows the dark and light phases during the LD cycles, during DD cycles no light is present. The number of days are on the Y-axis. (ZIP) [file pone.0169644.s003.zip › Supplementary material/S3 DR5 activity and Tb.tif]

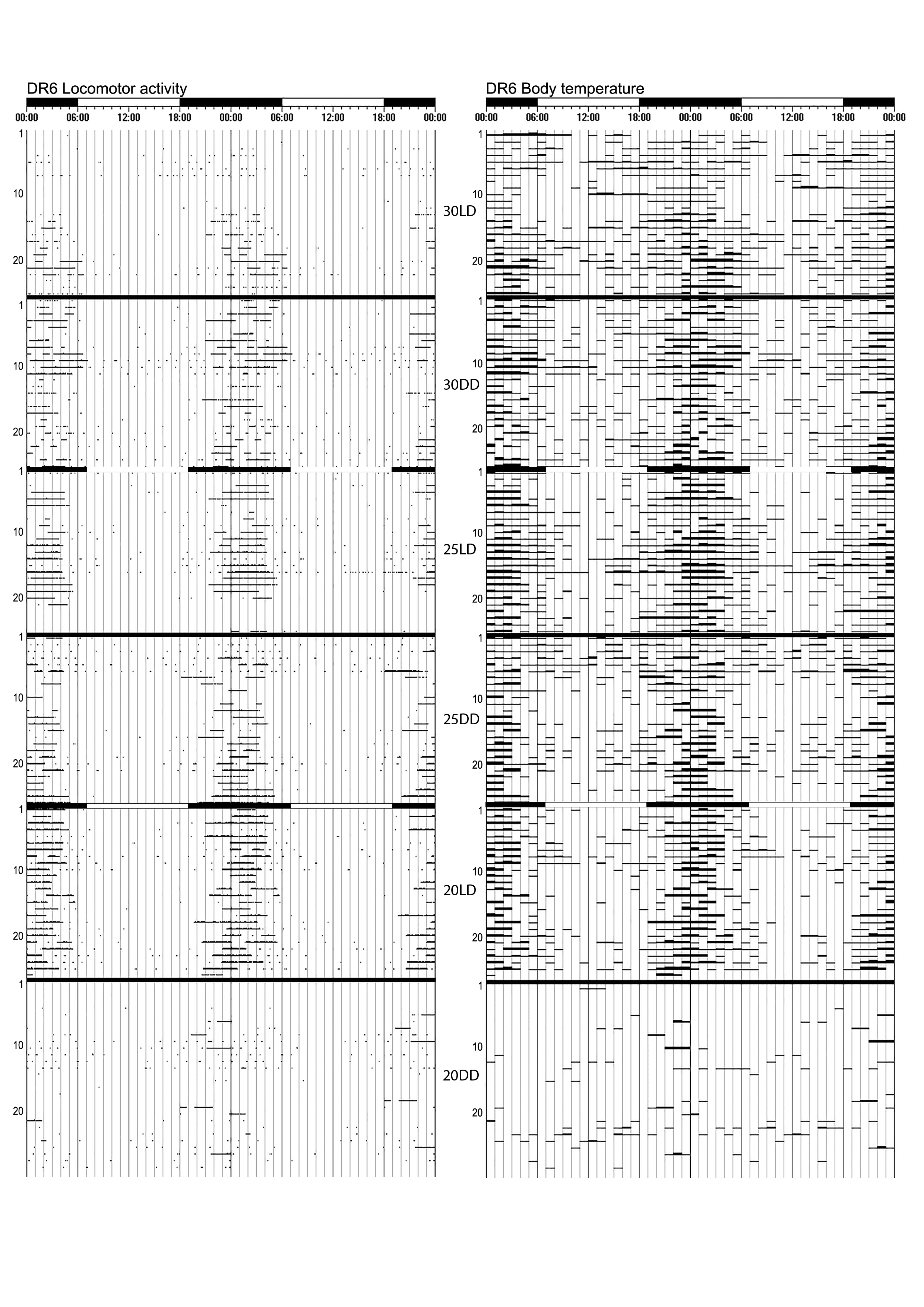

Supplement: S3 Fig — Complete actograms for the duration of the experimental procedure are presented for all animals. The black and white bars on top of the actograms shows the dark and light phases during the LD cycles, during DD cycles no light is present. The number of days are on the Y-axis. (ZIP) [file pone.0169644.s003.zip › Supplementary material/S3 DR6 activity and Tb.tif]

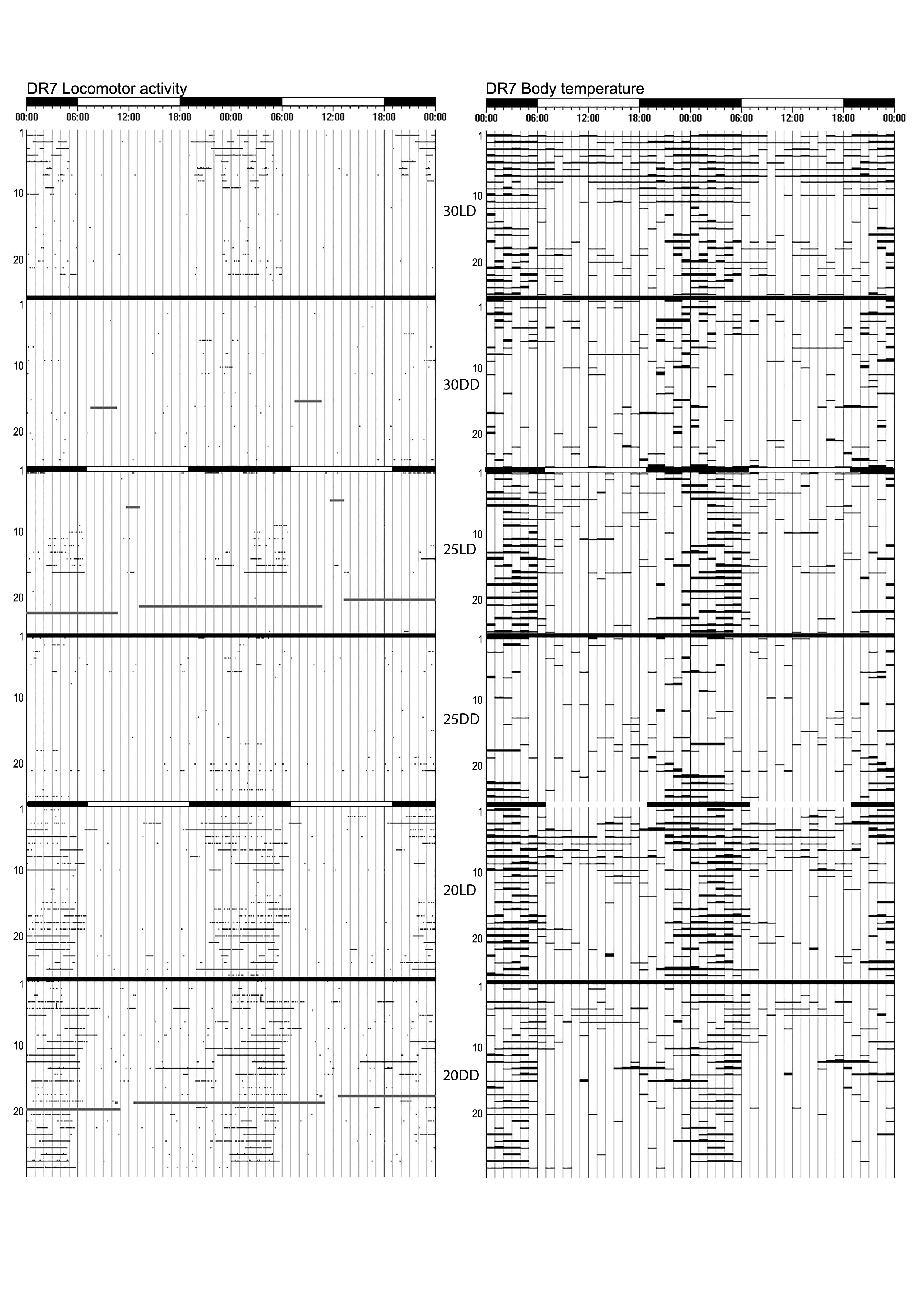

Supplement: S3 Fig — Complete actograms for the duration of the experimental procedure are presented for all animals. The black and white bars on top of the actograms shows the dark and light phases during the LD cycles, during DD cycles no light is present. The number of days are on the Y-axis. (ZIP) [file pone.0169644.s003.zip › Supplementary material/S3 DR7 activity and Tb.tif]

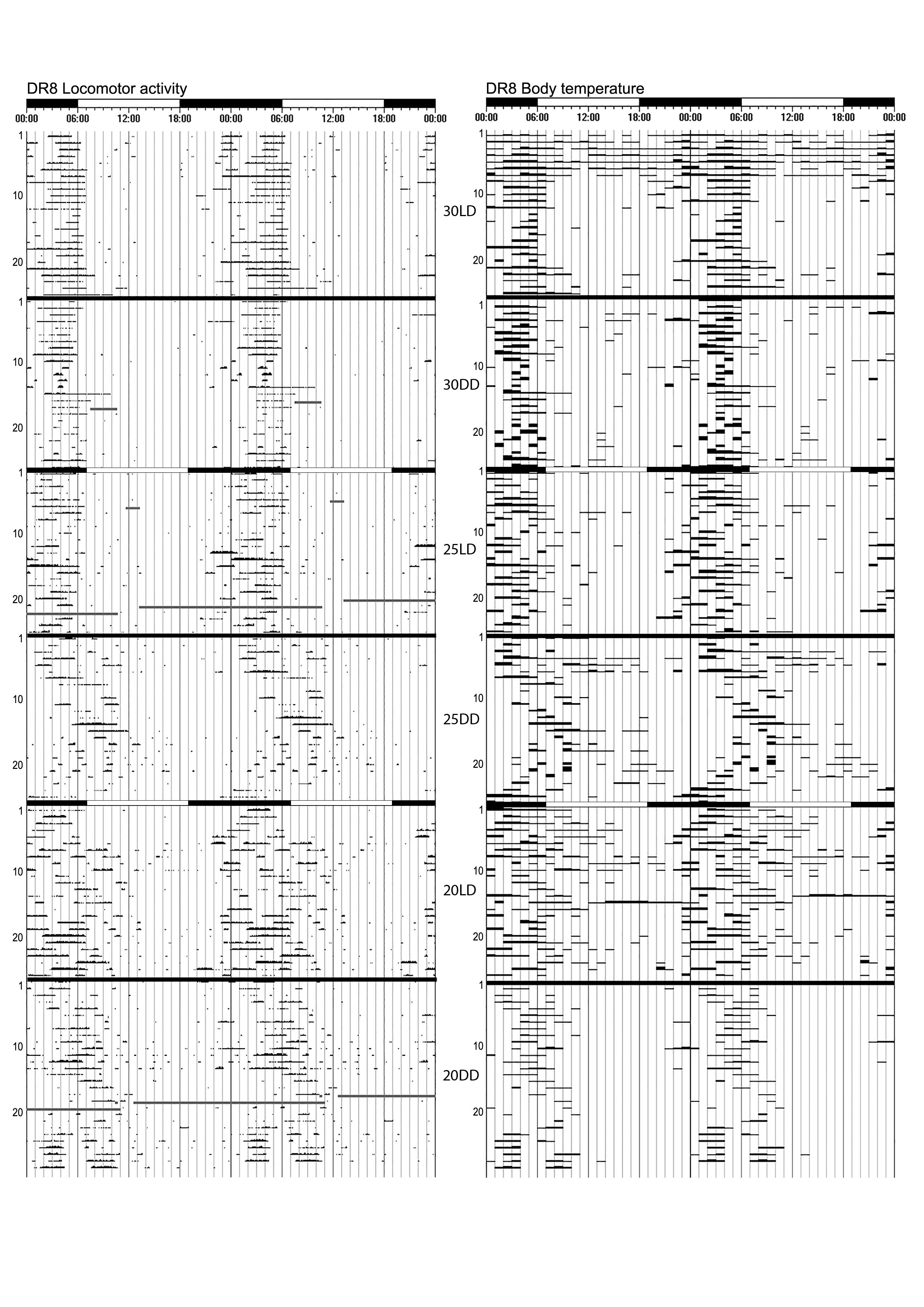

Supplement: S3 Fig — Complete actograms for the duration of the experimental procedure are presented for all animals. The black and white bars on top of the actograms shows the dark and light phases during the LD cycles, during DD cycles no light is present. The number of days are on the Y-axis. (ZIP) [file pone.0169644.s003.zip › Supplementary material/S3 DR8 activity and Tb.tif]

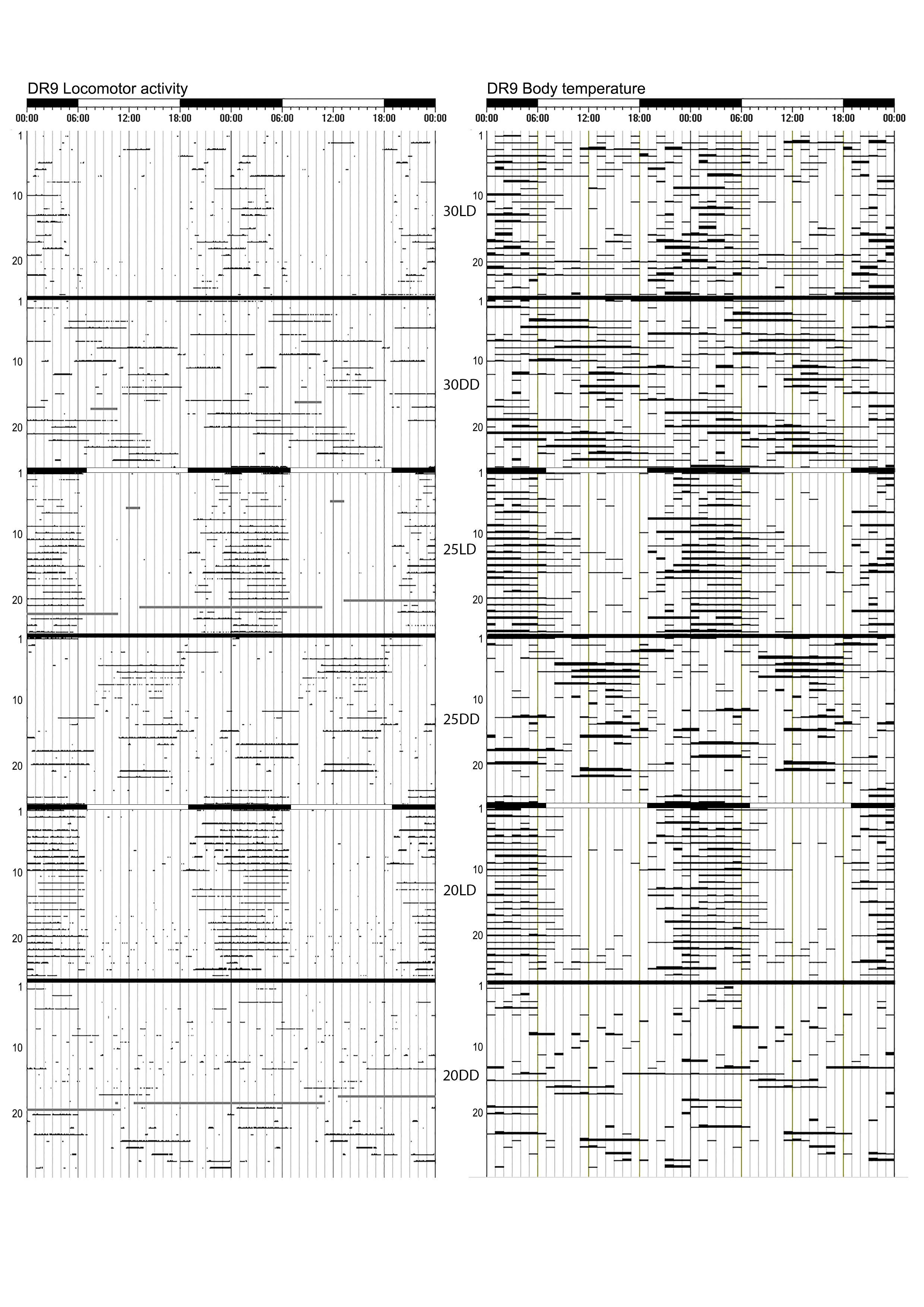

Supplement: S3 Fig — Complete actograms for the duration of the experimental procedure are presented for all animals. The black and white bars on top of the actograms shows the dark and light phases during the LD cycles, during DD cycles no light is present. The number of days are on the Y-axis. (ZIP) [file pone.0169644.s003.zip › Supplementary material/S3 DR9 act & Tb.tif]

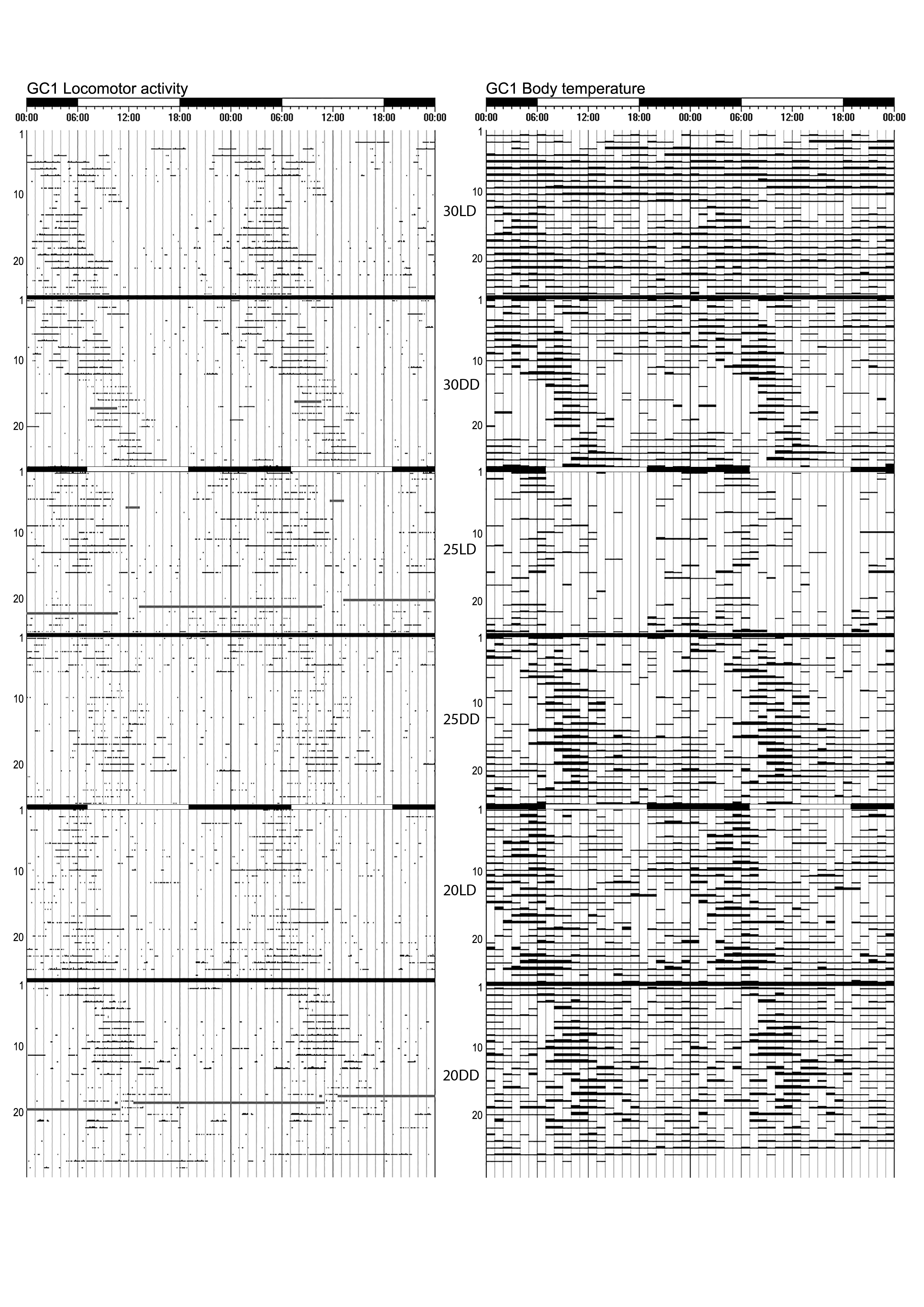

Supplement: S3 Fig — Complete actograms for the duration of the experimental procedure are presented for all animals. The black and white bars on top of the actograms shows the dark and light phases during the LD cycles, during DD cycles no light is present. The number of days are on the Y-axis. (ZIP) [file pone.0169644.s003.zip › Supplementary material/S3 GC1 activity and Tb.tif]

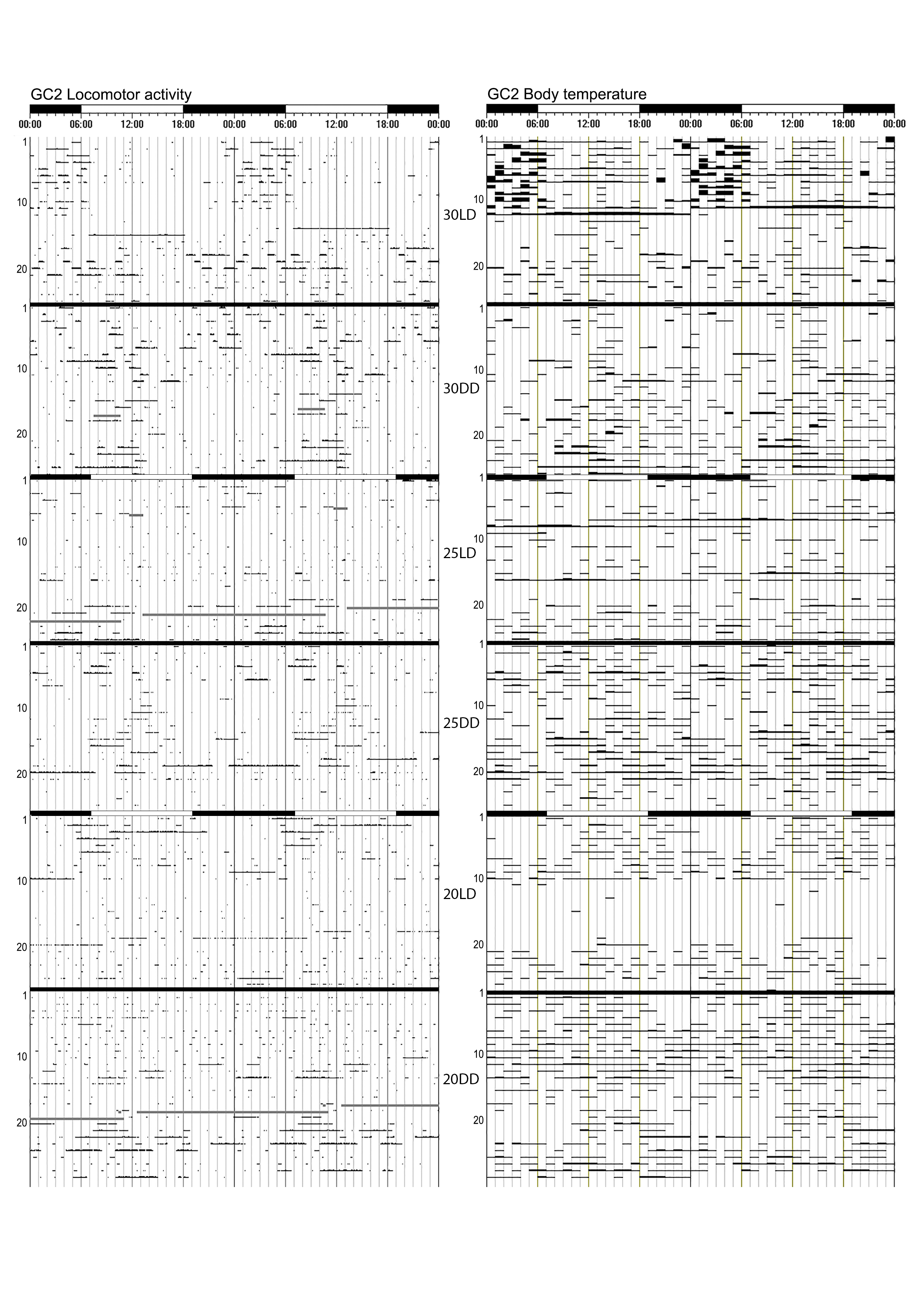

Supplement: S3 Fig — Complete actograms for the duration of the experimental procedure are presented for all animals. The black and white bars on top of the actograms shows the dark and light phases during the LD cycles, during DD cycles no light is present. The number of days are on the Y-axis. (ZIP) [file pone.0169644.s003.zip › Supplementary material/S3 GC2 act & Tb.tif]

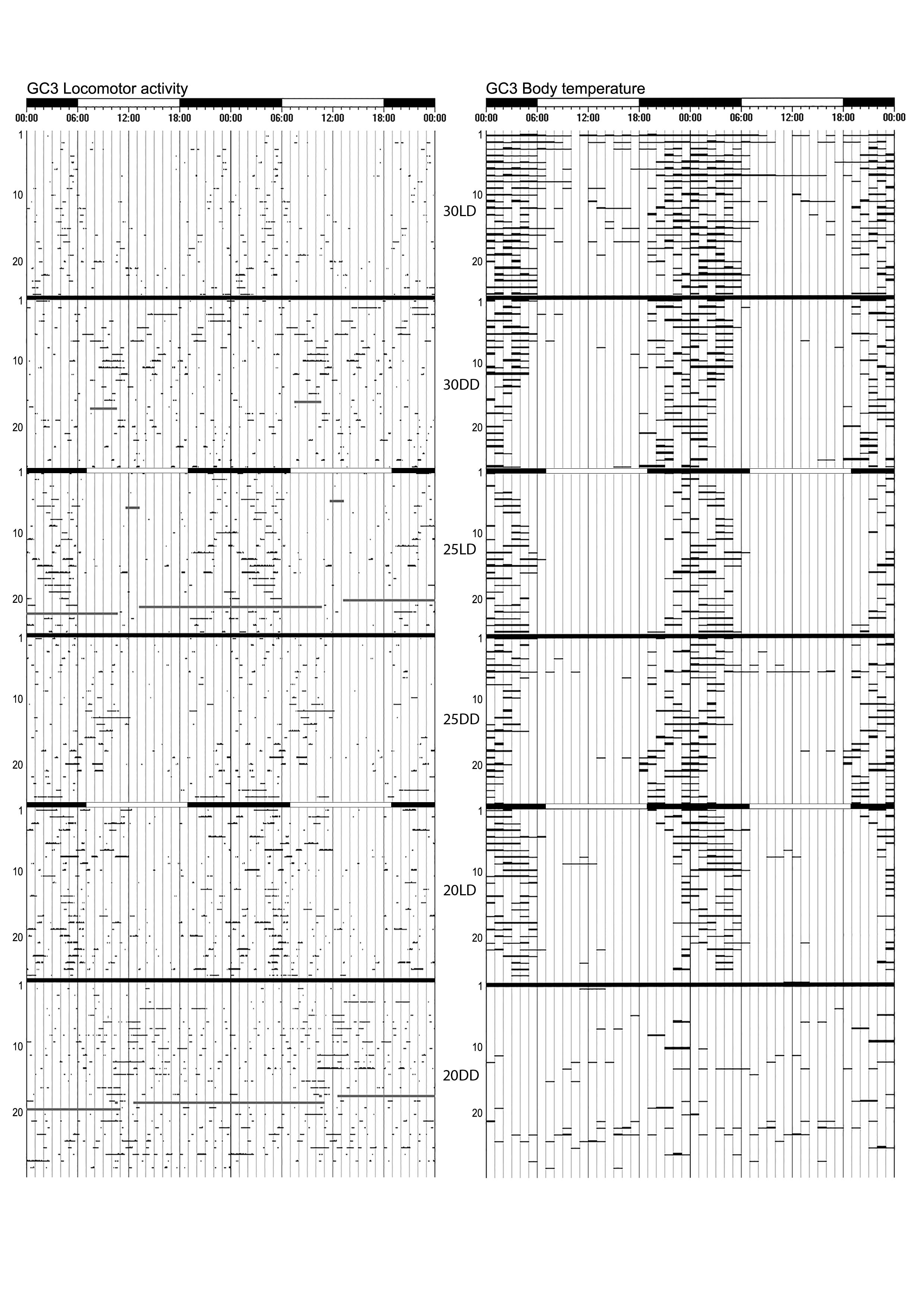

Supplement: S3 Fig — Complete actograms for the duration of the experimental procedure are presented for all animals. The black and white bars on top of the actograms shows the dark and light phases during the LD cycles, during DD cycles no light is present. The number of days are on the Y-axis. (ZIP) [file pone.0169644.s003.zip › Supplementary material/S3 GC3 activity and Tb.tif]

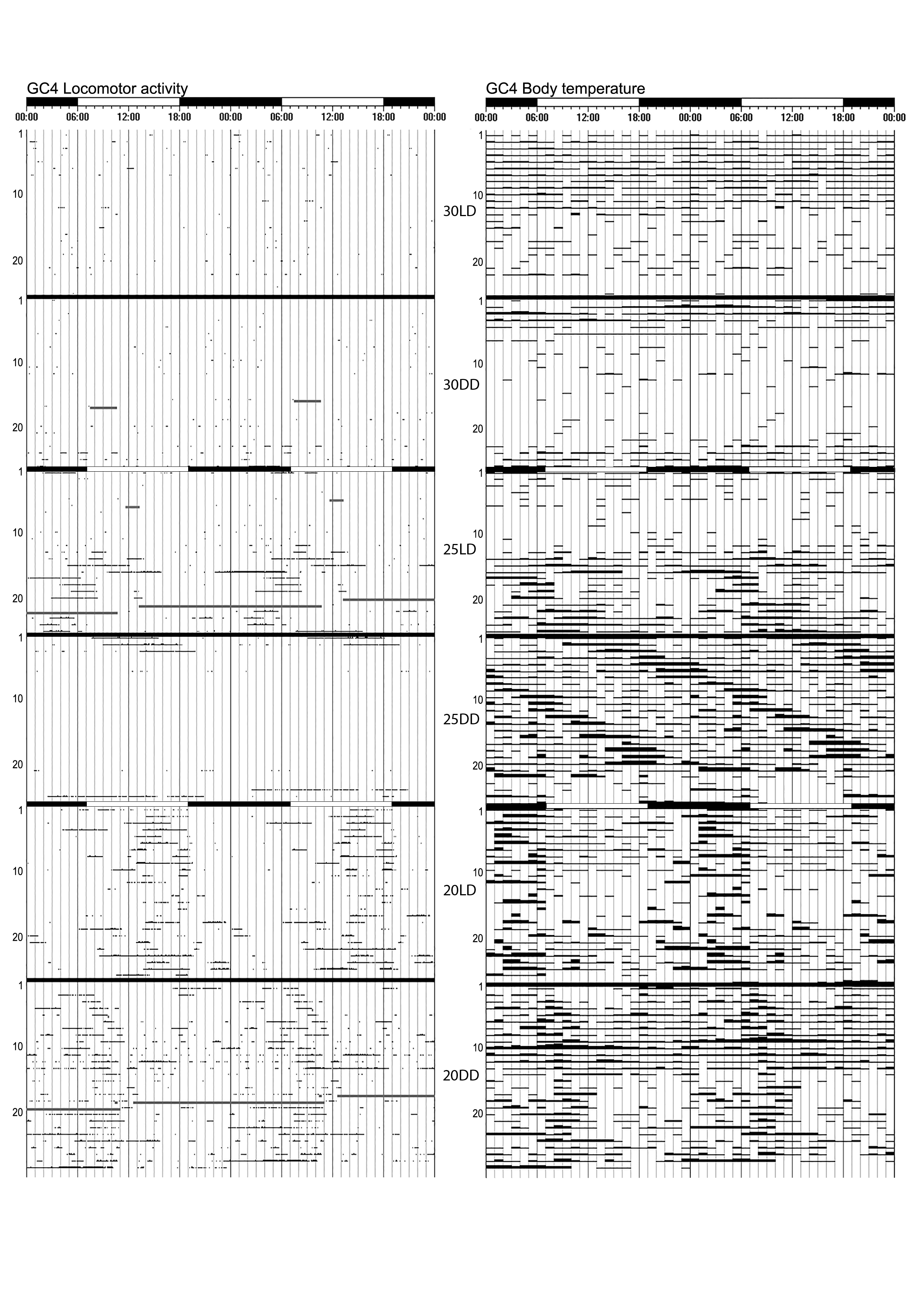

Supplement: S3 Fig — Complete actograms for the duration of the experimental procedure are presented for all animals. The black and white bars on top of the actograms shows the dark and light phases during the LD cycles, during DD cycles no light is present. The number of days are on the Y-axis. (ZIP) [file pone.0169644.s003.zip › Supplementary material/S3 GC4 activity and Tb.tif]

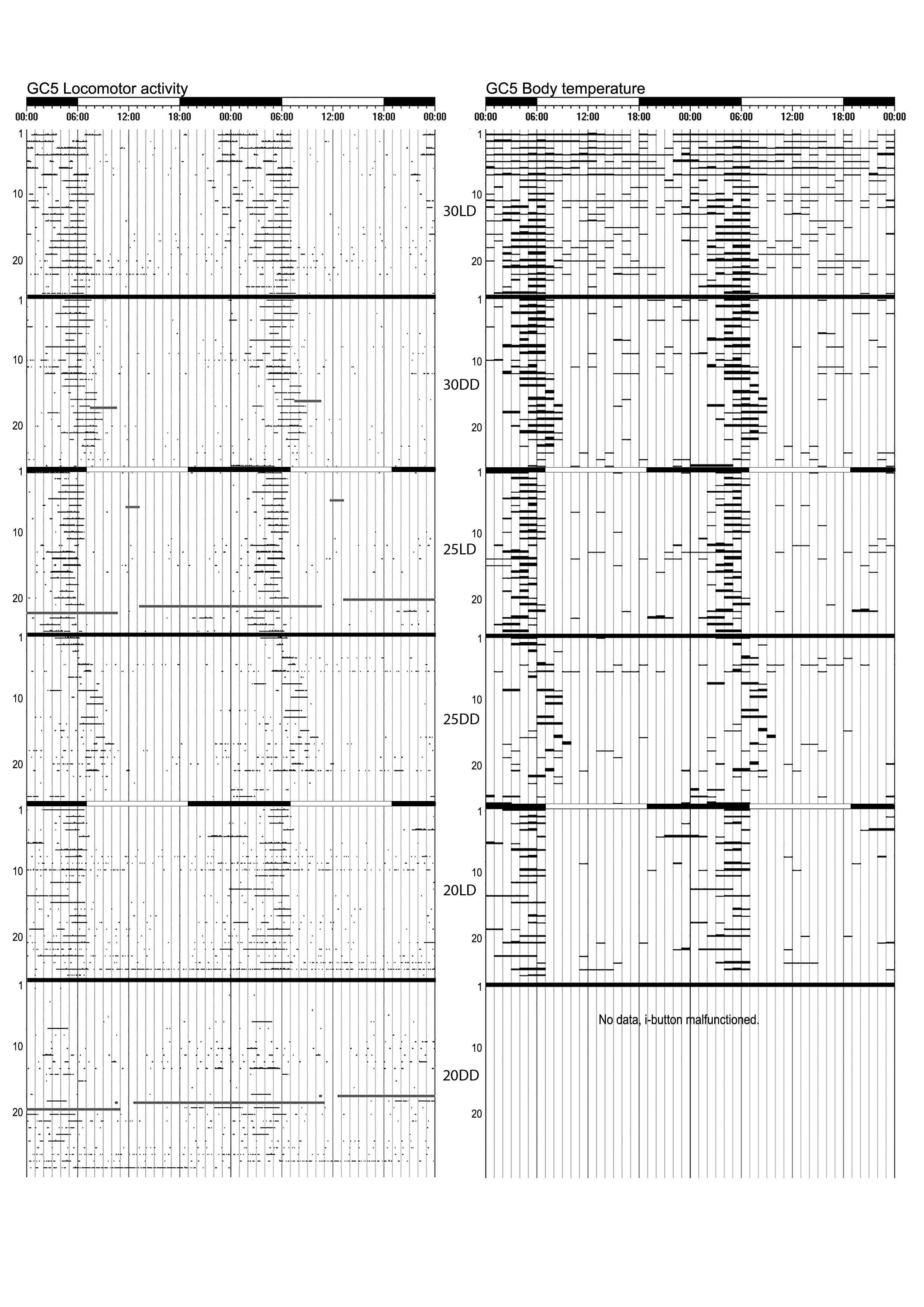

Supplement: S3 Fig — Complete actograms for the duration of the experimental procedure are presented for all animals. The black and white bars on top of the actograms shows the dark and light phases during the LD cycles, during DD cycles no light is present. The number of days are on the Y-axis. (ZIP) [file pone.0169644.s003.zip › Supplementary material/S3 GC5 activity and Tb_.tif]

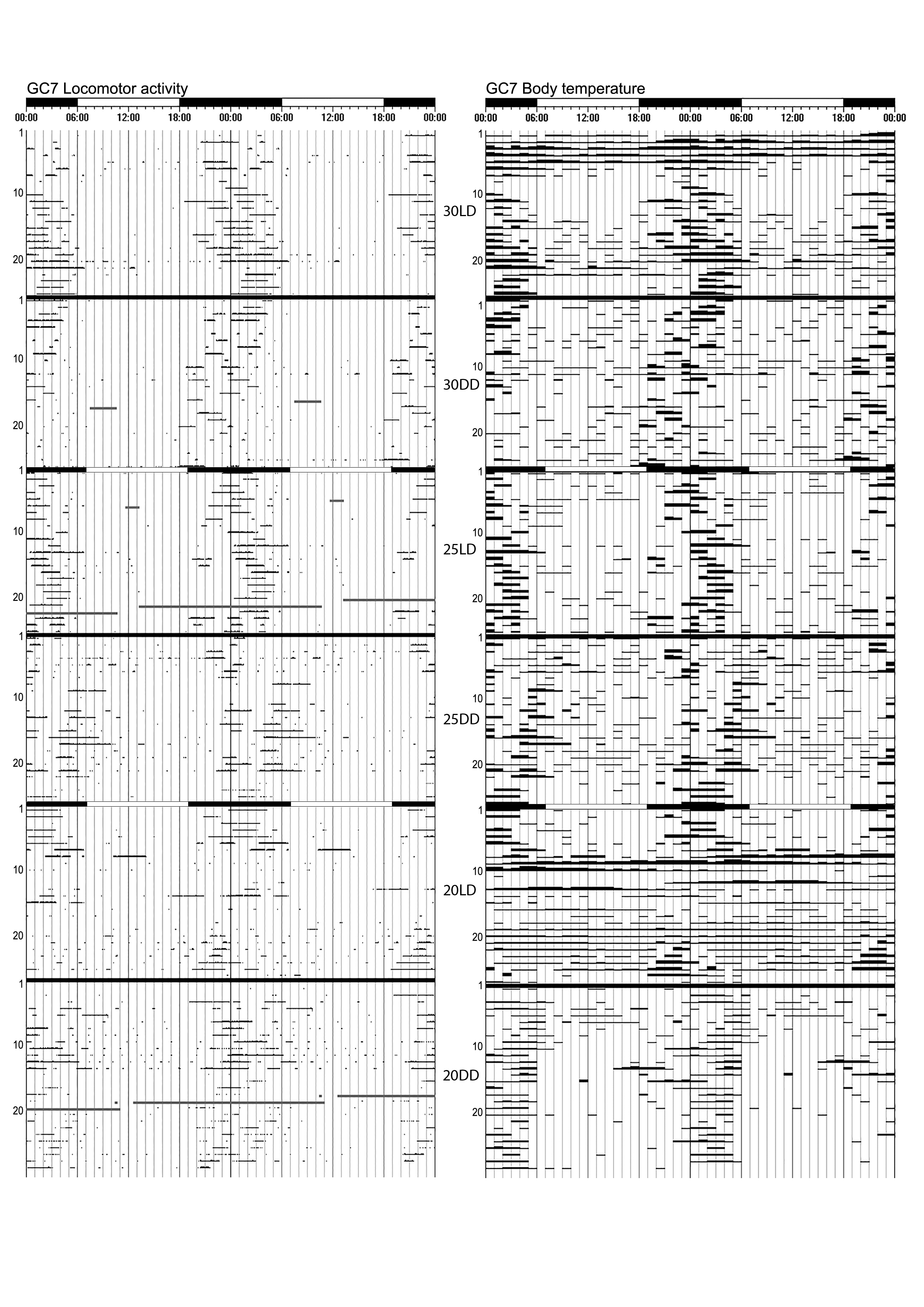

Supplement: S3 Fig — Complete actograms for the duration of the experimental procedure are presented for all animals. The black and white bars on top of the actograms shows the dark and light phases during the LD cycles, during DD cycles no light is present. The number of days are on the Y-axis. (ZIP) [file pone.0169644.s003.zip › Supplementary material/S3 GC7 activity and Tb.tif]
